# Postural Stability Changes During the 4 Phases of the Half Squat: Kinematics Profile of the Center of Pressure and Center of Mass in High-Performance Weightlifters—A Pilot Study

**DOI:** 10.3390/bioengineering13060711

**Published:** 2026-06-21

**Authors:** Emilio Manuel Arrayales-Millán, Miguel Rodal, Mirvana Elizabeth González-Macías, Carlos Villa-Angulo, Karla Raquel Keys-González, Arnulfo Ramos-Jiménez, Isabella Arrayales-Mejia, Kostantinos Gianikellis

**Affiliations:** 1 Laboratory Biomechanics, Faculty of Sports, Autonomous University of Baja California, Mexicali 21289, BC, Mexico; 2 BioErgon Research Group, University of Extremadura, 10003 Caceres, Spain; 3 Laboratory of Bioinformatics and Biophotonics, Engineering Institute, Autonomous University of Baja California, Mexicali 21289, BC, Mexico; 4 Laboratory of Exercise Physiology, Biomedical Sciences Institute, Autonomous University of Ciudad Juárez, Chihuahua 32310, CHH, Mexico; 5 Department of Biomechanics, University of Nebraska at Omaha, Omaha, NE 68182, USA

**Keywords:** Power-Based Training, mechanical demand, center of pressure, center of mass, total center of pressure, postural control, stability

## Abstract

This study investigated balance control during the half squat by analyzing the relationship between the center of mass (CoM) and the center of pressure (CoP) in five experienced male weightlifters performing segmented squats at five load levels (20–80% 1 RM) across four Power-Based Training (PBT) exercises. The area of the 95% confidence ellipse was quantified using the Vicon motion capture system in conjunction with AMTI force plates. Given the small sample size (*n* = 5), a dual inference approach was implemented—frequentist repeated-measures analysis of variance (ANOVA) complemented by a unified adaptive Bayesian hierarchical model—to mitigate Type II error in low-power scenarios. Regarding the movement phase, a marked effect on center of pressure (CoP) stability was observed, as evidenced by both statistical approaches (frequentist: F(1.65, 6.59) = 19.44, *p* = 0.002, ηp^2^ = 0.829; Bayesian: P(β_phase < 0) > 0.999). Although external load did not reach statistical significance in the frequentist analysis (*p* = 0.177, achieved power = 0.27), the Bayesian model provided moderate evidence of a positive impact (β_load = 0.059, 95% HDI [0.005, 0.115], *p* = 0.981). The area of the center of mass (CoM) ellipse showed no effects of interest. Limb asymmetries were significant and consistent throughout the experiment (frequentist: 48.01 ± 30.13%; Bayesian: 69.48%, 95% HDI [55.86%, 81.44%], P(AI > 20%) = 1.000) and were not modulated by the experimental condition. CoP-CoM coupling was stronger in the mediolateral direction than in the anteroposterior direction. The findings reveal that phase is the primary factor in postural stability, exerting a modest positive influence discernible only through low-powered probabilistic inference, and that the dual framework strengthens inferential robustness in small-sample biomechanical studies. Confirmatory studies with larger samples are recommended.

## 1. Introduction

Postural Control is a vital aspect of human movement, particularly during dynamic strength exercises such as the half squat, where stability must be maintained while handling significant external loads. Balance control regulates the interaction between the center of pressure (CoP) and the center of mass (CoM) during these activities. In this manuscript, the following abbreviations are used: total center of pressure (CoPt), left-foot center of pressure (CoPpi), right-foot center of pressure (CoPpd) and center of mass (CoM).

The fundamental variables in postural control research are the CoP and the CoM, where the CoP is reflected as the neuromuscular regulation of ground reaction forces, and the CoM represents the integrated kinematic result of the segmental movement of the whole body [1]. Conventional biomechanical studies of the half squat have focused mostly on joint kinetics and kinematics, but current methodologies highlight the importance of incorporating stability parameters to improve understanding of the relationship between mechanical response and postural control [2].

In the standing position, postural control employs an inverted pendulum model, in which the CoP and CoM each receive a signal proportional to the CoM’s horizontal acceleration, which the postural control system recognizes as an error signal [1]. This model is helpful for static postures and walking, but it does not fully explain the biomechanical problems that come up during a half squat.

The CoM is translated both vertically and horizontally during the half squat due to coordinated flexion and extension of the ankle, knee, and hip [3]. Adding a rigid mass (Olympic bar) to the body system (torso and limbs) changes its initial inertial properties. Therefore, the postural control strategy shifts from predominantly distal control (ankle strategy, which is predominant in still standing) to predominantly proximal active control (hip and trunk strategy), reflecting the increased neuromuscular demands of loaded dynamic movement [4,5]. The CoP is in accordance with Palmieri et al. [4], representing both the path of the CoM and the torque exerted at the support surface to control the body’s mass acceleration. This is important because an external load changes the distribution of moments throughout the joints, making the trunk and the bar behave as a unit. It has been demonstrated that athletes trained in a particular sport can alter their neuromuscular strategies to maintain stability under increasing external loads, with load-dependent changes in several CoP measures, including the variability and velocity of the CoP trajectory, although these patterns might differ between powerlifters and weightlifters [6].

Strength training exercises like the half squat are often used in sport training and rehabilitation because they effectively target strength, coordination, and posture. Prior biomechanical research, particularly that employing Power-Based Training (PBT), has demonstrated that the movement of the CoP, the quantity of mechanical power generated, and the distribution of joint mechanical power vary considerably across the specific phases of the half squat and the weight used [2].

In the literature, the half squat has been subdivided into four phases in the PBT framework [2], initially created for the analysis of mechanical power but later adapted for the analysis of the specific interactions between the CoP and the CoM [6,7]. More specifically, Giustino et al. [6] used phase-segmented analysis to compare postural control between powerlifters and weightlifters, and Rodal et al. [2] used the PBT framework to analyze CoM kinematics and joint powers [2]. The current PBT framework addresses the large change from eccentric to concentric (P2–P3) phases in countermovement activities. This enables us to distinguish the anticipatory (P1), braking (P2), propulsive (P3), and terminal (P4) phases of postural control [7].

The weighted squat is a biomechanically complex action due to both the forces acting on the joints and the need to maintain balance. Recent factor analyses have shown that during the high-bar squat, moments at the hip, knee, and ankle simultaneously influence the anteroposterior movement of the CoM [8]. In this case, ankle internal rotation, hip flexion–extension, and knee flexion–extension contribute similarly to CoM control, highlighting the multi-joint nature of postural regulation [8].

The CoP shows how the brain and muscles work together to maintain balance, complementing the mechanical information from the CoM [1,9]. Therefore, investigating how the center of mass changes across different movement stages and under varying loads can provide insights into how stability is regulated during high-intensity strength training.

The PBT framework offers a robust methodology for dividing movement into physiologically significant stages, based on the mechanical power exerted by the center of mass (CoM) [2]. By dividing the half squat into four phases, PBT enables analysis of each movement segment in relation to joint mechanical power and, potentially, postural stability. This separation is particularly relevant given the significant differences in neuromuscular demands, joint contributions, and balance control strategies between the phases [2]. Therefore, analyzing the CoM’s behavior patterns during the different phases of the half squat under varying loads is crucial for improving specific training methods and optimizing injury-prevention techniques in strength and conditioning programs. Specifically, analyzing the kinematics and kinetics of the CoM throughout the different phases reveals the complex relationship between mechanical load and the neuromuscular requirements of dynamic movements adapted to varying loads [2].

The neuromuscular mechanisms underlying stabilization during the loaded dynamic squat remain unclear, particularly regarding how CoP modulation interacts with CoM acceleration under variable mechanical demands. Traditional whole-cycle analyses are unable to capture the phase-dependent nature of these demands, as force production, braking, and stabilization place distinct requirements on the postural control system. To address this limitation, the present study employs a phase-segmentation strategy within the PBT framework, enabling us to specifically examine whether postural stability is regulated uniformly or if it exhibits phase-dependent sensitivity to variable loads. The CoP directly represents the neuromuscular control strategies used to regulate balance, reflecting the dynamic interaction between the neuromuscular system and the ground. This is evident in dynamic sports tasks, where expert athletes actively modulate CoP excursion to compensate for variations in the CoM and maintain stability [10]. The displacement of the center of mass encapsulates the integrated kinematic result of the entire segmental body movement, while the trajectory of the center of pressure (CoP) shows the neuromuscular control actions inherent in regulating said movement and maintaining dynamic equilibrium [1]. Furthermore, even in bilateral tasks, such as squat, asymmetric contributions of the lower limbs to postural control have been reported. Suggesting a potential functional specialization between the limbs [11,12].

In the sports domain, CoP trajectories can be used to assess postural stability via advanced analysis techniques, thereby supporting the use of CoP displacement measurements to monitor and enhance athletic performance [13].

Consequently, this investigation aimed to build upon prior biomechanical assessments grounded in the PBT by integrating stability metrics derived from the CoP, quantified through confidence ellipse areas, during the half-squat exercise. The 95% confidence ellipse area, a two-dimensional representation of CoP dispersion, has been widely used to assess postural stability in biomechanical and clinical research [4]. This method is based on the covariance matrix of the signal’s mediolateral and anteroposterior coordinates, with the semi-major axes of the ellipse indicating the directions of greatest and least CoP dispersion [14]. The 95% ellipse is also used to assess postural stability across different situations and to detect alterations in postural control under different experimental conditions [14,15]. This way, the CoM data obtained from the capture system are treated as two-dimensional paths for each phase and load. This allows a comparison of CoP, a neuromuscular control variable, with CoM, a global mechanical variable.

Modifying the CoP position during the squat significantly alters knee and ankle extensor moments and muscle activation [16]. This capacity to redistribute mechanical load through a simple visual cue makes this variable an effective tool for optimizing rehabilitation and training protocols [16]. Given substantial interindividual differences in kinematic and kinetic responses to increased load, experienced lifters often employ technical approaches to manage high joint torques during demanding lifts [17]. Therefore, it is crucial to consider individual movement adaptations when optimizing strength-training prescriptions [18].

The present study was guided by three primary objectives: 1. to examine how external load and different movement phases affected the total CoP (CoPt); 2. to identify any differences in CoP behavior between left (CoPpi) and right (CoPpd) legs; 3. to compare CoP responses with CoM dynamics. This comparison aimed to investigate a possible dissociation between the mechanical demands of the task and the postural control mechanisms used.

Given the exploratory nature and small sample size (*n* = 5), the following hypotheses were formulated: (H1) the CoP ellipse area will differ significantly across movement phases, reflecting phase-specific postural demands; (H2) the area of the ellipse at the center of pressure will show less impact from the external load compared to the phase of movement, demonstrating the predominance of phase-dependent neuromuscular demands over the magnitude of the load; (H3) the area of the ellipse at the center of mass will fluctuate descriptively according to the load and phase, but the statistical inference will be constrained by the sample size. Given the exploratory nature of this pilot study and the inherent limitations in recruiting elite weightlifters (*n* = 5), a dual inferential framework was adopted that combines frequentist and Bayesian methods to maximize inferential robustness under low statistical power [19].

## 2. Materials and Methods

### 2.1. Participants

Five male weightlifters from the Mexican national team, all national medalists in their respective bodyweight categories (U17, U20 y U23), volunteered for this study. The sample size of *n* = 5 reflects the inherent difficulty in recruiting elite competitive weightlifters willing to perform loaded maximal effort squats under laboratory conditions. The repeated measures design featuring 20 within-subject conditions (5 loads × 4 phases) provides 100 observations per variable, which partially offsets the small sample size [19,20]. None of the participants had musculoskeletal injuries at the time of the test and provided written informed consent prior to participation. The experimental protocol was approved by the institutional ethics committee and was conducted in accordance with the Declaration of Helsinki [21]. Powerlifters and weightlifters demonstrate distinct postural control patterns during loaded squats, adopting different strategies in response to mechanical demands [6].

The same cohort of participants and the same experimental protocol were previously described in detail by Rodal et al., who analyzed center-of-mass kinematics and joint mechanical power [2,7]. The present study builds on this validated protocol; however, all outcome variables (CoP and CoM ellipse areas, interlimb asymmetry indices, and CoP–CoM correlations), statistical analyses, and interpretations presented here are novel and do not overlap with those prior reports.

### 2.2. Evaluation Protocol

Participants performed the half-squat exercise under five external load conditions corresponding to 20%, 35%, 50%, 65%, and 80% of their one-repetition maximum (1 RM). Load increments were selected to cover the functional training range from light technical work (20% 1 RM) to near-maximal loads (80% 1 RM), with 15% intervals chosen to balance physiological resolution with the practical limitations of managing fatigue in a single session. This range is consistent with that reported by Swinton et al. [17]. Each athlete performed five sets with a pre-established load progression (20%, 35%, 50%, 65%, and 80% of 1 RM), involving five repetitions per load and rest periods of three to five minutes to mitigate accumulated fatigue. This stepped load methodology was implemented for three reasons: 1. safety, considering that near-maximal loads were attempted only after adequate neuromuscular warm-up at lower intensities; 2. uniformity of technique, given that the partial squat demands a meticulous movement pattern that is progressively consolidated from submaximal to maximal efforts; 3. competitive dynamics, since weightlifters usually employ ascending load progressions during their training and competitions, making this sequence environmentally relevant for the study population. The test–retest reliability of the elliptical area measurements was not investigated over several days, with this factor constituting an inherent restriction of the single-session conception (see the Limitations section).

Each repetition was performed under controlled conditions, with standardized foot placement and support distance, and adequate rest was provided between trials to minimize fatigue effects, as previously described [2]. The same kinematic (Vicon) and kinetic (AMTI) acquisition protocols, filtering parameters (fourth-order Butterworth, 6.5 Hz cutoff), and phase detection algorithms were applied uniformly across all participants and loading conditions to ensure procedural consistency [2].

**Figure 1:**
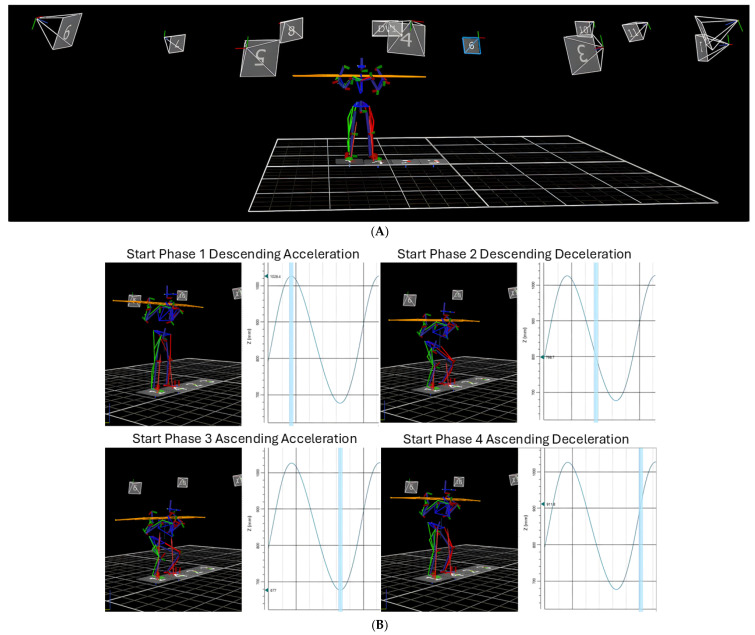
(**A**) Arrangement of cameras and force plates Biomechanics Laboratory and (**B**) four-phase PBT Framework. Note: (**A**): 12-camera Bonita 10 Vicon (11 optoelectronic cameras and 1 video camera) and 2 force plates AMTI ORC-6-2000; (**B**): Phase 1: Descending acceleration, Phase 2: Descending deceleration, Phase 3: Ascending acceleration, Phase 4: Ascending deceleration.

The half squat was segmented into four biomechanical phases within the PBT framework, based on kinematic and kinetic criteria, following the methodology proposed by Rodal et al. [2]: P1: Descent acceleration (start to maximum negative velocity); P2: Descent deceleration (maximum negative velocity to zero velocity at bottom); P3: Ascent acceleration (bottom to maximum positive velocity); P4: Ascent deceleration (maximum positive velocity to end, zero velocity) (see Figure 1A). Phase transition points were detected algorithmically using the vertical velocity of the CoM: zero-crossing was defined as the first frame in which the vertical velocity changed sign, with a tolerance of ±0.05 m/s to avoid noise. All kinematic data were low-pass filtered using a fourth-order Butterworth filter with a cutoff frequency of 6.5 Hz, as described in Rodal et al. [2]. This segmentation allowed independent analysis of the CoP stability metrics within each biomechanically distinct phase. The phases described correspond to functional parts of the movement and are characterized by changes in the system’s velocity and acceleration.

### 2.3. Data Acquisition

Ground reaction forces were recorded using AMTI force platforms [22] at a sampling rate of 1000 Hz, enabling the high-resolution measurement of the CoP coordinates. Whole-body kinematics were captured using a Vicon motion capture system [23] operating at 250 Hz, with reflective markers positioned according to the Vicon full-body markers Plug-In Gait [24], in conjunction with Vicon Nexus software version 2.16 [25], all of which have been previously validated [2] (see Figure 1B).

Left- and right-foot displacement trajectories (CoP), feet together (CoPt), and CoM were used only as intermediate inputs for covariance matrix and 95% confidence ellipse calculations. Displacement trajectories were not analyzed as independent kinematic variables; they were used just to quantify spatial dispersion and postural stability for each load-phase combination. Force and kinematic data were synchronized and filtered following the procedures described in Rodal et al. [2,7].

### 2.4. Calculation of the Center of Pressure and the Center of Mass

The CoP was calculated from the force platforms using the ground reaction forces. The total CoP is the weighted average of the left and right CoPs, based on vertical forces. For Python execution, linear algebra functions were used to calculate eigenvalues (numpy.linalg.eigvals or numpy.linalg.eigh), and scipy.stats.chi2 was used. ppf (0.95, df = 2) was used for the quantile.

The CoM trajectory was calculated using kinematic data and a 14-segment anthropometric model (head, trunk, pelvis, bilateral upper arms, forearms, thighs, shanks, and feet) with segmental masses and center of mass locations derived from the Vicon Plug-In Gait marker set and the anthropometric tables of Dempster (1955) [26], as previously validated [2]. The CoM coordinates were calculated for each instant and subsequently segmented according to the load condition and phase of motion.

### 2.5. Analysis of the Area of the Confidence Ellipse

The 95% confidence ellipse area was selected as the primary postural stability metric for three important reasons. First, the ellipse area, unlike path-based metrics such as total path length or mean velocity, is unaffected by the duration of the analyzed span and encompasses the spatial dispersion of the CoP/CoM regardless of movement speed [14,15]. This becomes very important in segmented phase analyses where phases vary greatly in duration. Second, the ellipse area merges mediolateral and anteroposterior variability into a single unit, resulting in a very comprehensive two-dimensional stability index [14]. Third, frequency metrics such as RMS amplitude and center frequency assume stationarity that may not hold true during dynamic squats, but the ellipse area does not require assumptions about the temporal structure of the signal [15].

Postural stability was quantified using the area of the ellipse with 95% confidence, calculated from the mediolateral and anteroposterior coordinates of the CoP and CoM, using the formula Area = π × χ^2^_0_._95,2_ × √(det(Σ)), where χ^2^_0_._95,2_ = 5.991 and det(Σ) is the determinant of the covariance matrix [14,15]. Ellipse areas were calculated separately for the total CoP (CoPt), left-foot CoP (CoPpi), right-foot CoP (CoPpd), and the CoM. Ellipse areas were log-transformed (base 10) before statistical analysis to normalize the distribution and stabilize variance [15]. All repeated-measures ANOVAs were performed on the log-transformed values. For clarity, figures display geometric means on logarithmic scales; error bars represent standard errors derived in the log-domain and back-transformed appropriately. For each participant and load-phase combination, the ellipse areas were averaged across repetitions and are reported in square meters (m^2^). The inter-limb asymmetry index (AI) of ellipse areas for each foot was defined as AI = |CoPpi − CoPpd|/(0.5 × (CoPpi + CoPpd)) × 100 [11,12]. High asymmetry was classified as ≥20%, a threshold commonly applied in postural studies.

Data Exclusion Criteria. Data quality was assured by objective exclusion criteria (ellipse area <10^−6^ m^2^, inter-limb ratio >10, CoP trajectory jumps >5 mm between consecutive frames, non-finite values, or fewer than 30 valid samples). No trials met any exclusion criterion; all 100 expected measurements (5 participants × 5 loads × 4 phases) were retained. Extreme asymmetry values (>100%) were preserved, as they may reflect legitimate functional strategies in elite weightlifters. A complete validation log is provided in Table A3.

### 2.6. Statistical Analysis

The following analyses were performed under each framework:

Frequent Analysis: A two-way repeated measures ANOVA with Greenhouse–Geisser correction was used for all dependent variables, such as CoPt, CoPpi, CoPpd, the ellipsoidal areas of CoM, and the skewness index. Post hoc pairwise comparisons were performed with Bonferroni correction, Shapiro–Wilk normality tests, and Mauchly tests of sphericity. Reliability between replicates was assessed using the intraclass correlation coefficient ICC(2,1); while Pearson correlation coefficients between CoP and CoM trajectories were tested using Steiger’s Z test for dependent correlations [26,27,28,29].

Effect sizes are presented as partial eta-squared (ηp^2^) along with 95% confidence intervals for all main effects and ANOVA interactions. Cohen’s d is also used for all paired post hoc comparisons [30].

Bayesian analyses: Hierarchical linear mixed-effects models were applied for CoPt, CoPpi, CoPpd, and CoM (logarithmically transformed); a logit-normal model for the skewness index; and a normal–normal conjugate update in Fisher’s Z-space for the CoP-CoM correlations. All Bayesian models made use of unified adaptive empirical priors, with a shrinkage of 0.8 towards anchors reported by the literature [31,32].

Statistical analyses were conducted with Python (Pingouin v0.5.5) [27,33]. The very small sample size (*n* = 5) led us to adopt a dual inferential framework: i) frequentist two-way repeated-measures ANOVA (RM-ANOVA) to maintain comparability to the established biomechanical literature, and ii) Bayesian hierarchical modeling to obtain probabilistic statements about effect existence and direction that are less sensitive to Type II errors under low power [19,20].

A two-way repeated-measures ANOVA was performed on the logarithmically transformed ellipse areas, using load (five loads: 20%, 35%, 50%, 65%, and 80% 1 RM) and movement phase (four levels: P1–P4) as within-subject factors. Given the small sample size, all within-subject effects were corrected using the Greenhouse–Geisser correction, regardless of the Mauchly test result. Effect sizes are presented as partial eta-squared (ηp^2^) with 95% confidence intervals; values of 0.01, 0.06, and 0.14 are considered small, medium, and large, respectively [30].

Hypothesis testing. The Shapiro–Wilk test was used to determine the normality of the log-transformed data for each load-phase combination (Table A1). Between-replicate reliability was calculated using the intraclass correlation coefficient (ICC) [28]. Correlations between the CoP and CoM. Pearson correlation coefficients between the CoP and CoM trajectories were calculated separately for the mediolateral (ML) and anteroposterior (AP) directions. The coefficients were transformed using Fisher’s Z-transform before aggregation. The 95% confidence intervals were constructed in the Z domain (SE = 1/√(*n* − 3)) and inversely transformed. A Z test for dependent correlations [29] was used to analyze the difference between the ML and AP correlations.

#### 2.6.1. Bayesian Approach

To overcome the limitations of null hypothesis significance tests for *n* = 5, the frequentist analysis was supplemented with hierarchical Bayesian models, using PyMC 5.16 [31] and ArviZ 0.19 [32]. This strategy allows for direct probabilistic statements about the hypotheses and is less prone to false negatives when power is low [20]. The Bayes factor (probability) is presented as *β* ± ε%, the credible interval as 95% of the high-density interval (95% HDI), and the size of the power as Cohen’s d.

Specification model. For each postural variable (CoPt, CoPpi, CoPpd, CoM), we fitted a linear mixed-effects model:

log_10_(Area*_ij_*) = *β*_0_ + ∑^4^*_k_*_=2_ *β_phase*·I(phase = *k*) + *β-load·*Load_scaled + *β_load*^2^·Load^2^_scaled + *β_interaction·*Phase·Load + *μ*_0*j*_ + ε*_ij_*

where Load_scaled is centered and scaled (μ = 0, σ = 1, corresponding to 20–80% of 1 RM), *μ*_0j_ ~ N(O, σ^2^_subject) represents random intercepts per participant, and ε*_ij_* ~ N(O, σ^2^_residual). Quadratic and interaction terms were included to capture possible nonlinear dose–response relationships.

Prior distribution. Unified adaptive empirical priors were employed for all Bayesian models (CoPt, CoPpi, CoPpd, CoM, and asymmetry), following the empirical Bayesian methodology [34,35]. This approach calculates prior hyperparameters directly from the observed data, then shrinks them toward the literature-informed anchors to prevent overfitting in small samples (*n* = 5).

Procedure. (1) For each variable, descriptive statistics (mean, SD) were computed for log-transformed ellipse areas across all conditions. (2) These empirical estimates were combined with the literature-informed anchors (intercepts: Schuber [15] and Quijoux [14]; phase effect sizes informed by Rodal [2,7] and Giustino [6]; load effects from Sohn [5] and Swinton [17]) via precision-weighted averaging: μ_combined = (μ_emp·τ_temp + μ_lit·τ_lit)/μ_emp·τ_lit), where τ = 1/σ^2^. (3) A shrinkage factor of 0.8 was applied to the empirical SD to avoid overfitting [35].

The resulting unified priors (shared across all regression models):

Intercept (*β*_0_) N(−3.5, 0.45) [log_10_(m^2^)].

Phase effects (*β*_phase): N(0, 0.25).

Load linear (*β*_load): N(0, 0.18).

Load quadratic (*β*_load^2^): N(0, 0.12).

Phase × Load interaction: N(0, 0.15).

Between-subject SD (σ_subject): Half-Cauchy(0.25).

Residual SD (σ_residual): Half-Cauchy(0.40).

For the asymmetry model (logit-normal), the prior for the intercept was α ~ 50% N(0, 1) on the logit scale, corresponding to ~50% asymmetry a priori.

For CoP-CoM correlations, subject-specific priors were estimated in Fisher-Z space from intra-individual variability, shrunk by 20%, and combined with global population priors [35,36]. Resulting priors: ML z ~ N(0.21, 0.56)[r ~ 0.21]; AP z ~ N(1.20, 0.51)[r ~ 0.83].

Post hoc calculation. Four MCMC chains of 2000 extractions each (8000 samples in total) were run using the No-U-Turn Sampler (NUTS). Convergence was considered to have occurred if the R-hat was less than 1.01 and the effective sample size (ESS) was greater than 1000 for all parameters [31,32]. Residual analyses and predictive checks (PPCs) conformed to a good model fit (RMSE ~ 0.39 for all models).

Interpretation criteria. Following Kruschke [19], posterior probabilities were classified as follows: *P*(effect > 0) > 0.99 indicates strong evidence for the effect direction; *P*(effect > 0) > 0.95 indicates a positive or negative effect (depending on the sign of the posterior mean); *P*(effect > 0) between 0.05 and 0.95 indicates uncertainty regarding the effect direction. A 95% highest density interval (HDI) that excludes zero was interpreted as a credible, non-null effect.

Asymmetry model. To respect the limits [0%, 100%], the limb asymmetry index was modeled with a logit-normal distribution:

logit(AI/100) = α + *β*_phase_ + *β*_load_ + ε

with the priors α ~ N(0, 1), *β_phase* ~ N(0, 0.5), and *β_load* ~ N(0, 0.3). Posterior predictions included *P* (AI > 20%|data) as a clinically relevant threshold for classifying substantial inter-limb differences [11,37].

Bayesian correlations between CoP and CoM. In each condition (subject × load × phase), we applied the conjugate normal–normal update in the Fisher-Z space. Posterior means and 95% HDIs were inversely transformed to the r scale. Global summaries aggregated the posterior distributions of the 100 conditions.

#### 2.6.2. Integration of Both Frameworks

The results are presented first using frequentist statistics (to facilitate comparison with the existing literature), followed by Bayesian summaries when the frequentist analysis is inconclusive due to low power. Confidence is strengthened when both approaches agree. In case of disagreement, the Bayesian interpretation is given priority because it yields better results with small samples [19,20]. All Bayesian analyses were performed on the same dataset used for the frequentist tests, ensuring internal consistency.

## 3. Results

### 3.1. Total Center of Pressure (CoPt)

**Figure 2:**
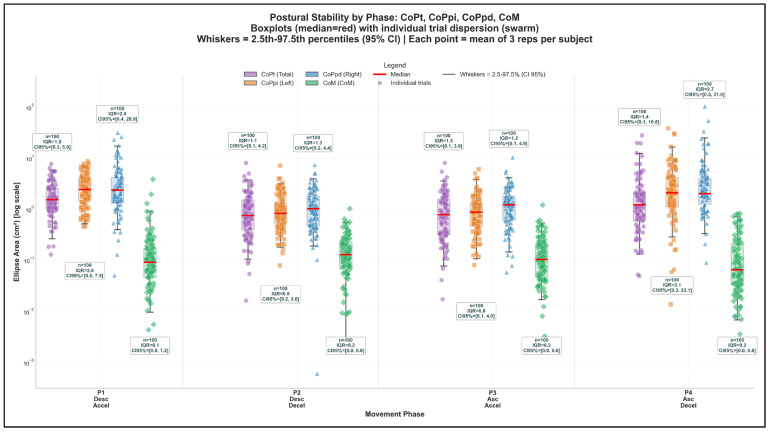
Postural stability across biomechanical phases. Boxplots (median = red line; whiskers = 2.5th–97.5th percentiles) with individual trial dispersion (swarm plots) for CoPt, CoPpi, CoPpd, and CoM ellipse areas. Each point represents the mean of 3 repetitions per subject (*n* = 100 per phase). Note the logarithmic y-axis.

Frequentist analysis. The repeated measures ANOVA on log-transformed CoPt ellipse area showed a highly significant main effect of the motion phase (F(1.65, 6.59) = 19.44, *p* = 0.002, ηp^2^ = 0.829, 95% CI [0.55, 0.91]). Post hoc comparisons with the Bonferroni correction indicated that Phase 4 (ascent deceleration) differed significantly from all other phases (all corrected *p <* 0.05) and exhibited the largest ellipse area (Figure 2). Phase 1 and Phase 3 did not differ significantly from Phase 2 after Bonferroni correction (corrected *p* = 0.095 and corrected *p* = 1.000, respectively).

The main effect of load did not reach statistical significance (F(1.27, 5.10) = 2.48, *p* = 0.177, ηp^2^ = 0.382, 95% CI [0.00, 0.71]). Notably, the confidence interval includes zero, indicating imprecise estimation compatible with a null effect but not precluding a moderate or even large effect. Thus, the absence of a significant load effect cannot be taken as evidence that load does not influence CoPt stability; the study lacked sufficient power to detect a moderate-sized effect. The statistical power achieved was 0.27, well below the conventional 0.80 threshold. The Load × Phase interaction was non-significant (F(1.75, 6.98) = 0.78, *p* = 0.479, ηp^2^ = 0.163) (see Figure 2).

Bayesian analysis. The hierarchy confirmed strong evidence for phase effects. The baseline intercept was credibly negative (β_0_ = −3.825 ± 0.115, 95% HDI [−4.044, −3.585], *p* < 0.001, Cohen’s d = −33.35), corresponding to a geometric mean area of 1.50 × 10^−4^ m^2^ in Phase 1. Phase contrasts showed strongly negative effects for Phases 2 and 3 relative to Phase 1 (Phase 2 vs. Phase 1: β = −0.285 ± 0.054, 95% HDI [−0.395, −0.181], P(β < 0) = 0.999, d = −5.28; Phase 3 vs. Phase 1: β = −0.313 ± 0.055, 95% HDI [−0.424, −0.210], P(β < 0) = 0.999, d = −5.69), indicating credibly smaller ellipse areas during deceleration on descent and acceleration on ascent. The Phase 4 contrast was uncertain (β = −0.090 ± 0.056, 95% HDI [−0.192, 0.123], P(β < 0) = 0.946) with the HDI including zero, consistent with the frequentist post hoc significance of Phase 4 but revealing that the posterior distribution is ambiguous rather than definitively negative.

For load, the linear coefficient was positive and credibly non-zero (β = 0.059, ± 0.028, 95% HDI [0.005, 0.115], P(β > 0) = 0.981, d = 2.07), indicating that each 30% increase in load corresponds to a +0.059 log_10(m_^2^_)_ increase in area (~+14.5% area). The quadratic term was uncertain but with high uncertainty (β = 0.029 ± 0.043, 95% HDI [−0.058, 0.110], P(β > 0) = 0.743). The Phase × Load interaction was negative but uncertain (β = −0.048 ± 0.025, 95% HDI [−0.097, 0.003], P (β < 0) = 0.968). Subsequent predictive checks confirmed that the model fit well (RMSE = 0.384, observed and predicted distributions aligned).

Integration. Both frameworks agree on a robust phase effect: frequentist *p* = 0.002 and Bayesian P(β < 0) > 0.99 for P2/P3 vs. P1. For load, the frequentist result was inconclusive (*p* = 0.177, CI includes zero), while the Bayesian HDI [0.005, 0.115] excludes zero for the linear term, providing moderate evidence that load exerts a positive effect on CoPt area. The Bayesian frameworks offer greater granularity: the phase effect is credibly negative for P2 and P3, while the P4 contrast is ambiguous despite frequentist post hoc significance, reflecting the conservative nature of the Bonferroni correction with small samples. The point estimate of the phase effect was 2.17 times that of the load effect (ηp^2^ = 0.829 vs. 0.382); however, the Bayesian load coefficient is credibly positive (P(β_load > 0) = 0.981), suggesting that the frequentist non-significance reflects low power rather than the absence of effect.

This divergence does not indicate an inherent opposition between statistical frameworks; on the contrary, it highlights their disparate sensitivity to effect size data. The frequentist *p*-value is subject to both effect size and sample size, while the Bayesian posterior distribution is primarily anchored in the likelihood of the observed data against rival hypotheses. With a sample size of *n* = 5, the frequentist test lacks the power to reject the null hypothesis, even for moderate effects; in contrast, the Bayesian model leverages prior information and the full posterior distribution to weigh probabilistic support [19,20]. Consequently, the Bayesian value P(β_load > 0) = 0.981 should be understood as an indication that a positive loading effect exhibits greater compatibility with the evidence than a null effect, and not as confirmation that such an effect is substantial or clinically relevant.

### 3.2. Center of Pressure per Foot (Left and Right CoP)

Left foot (CoPpi). The ANOVA revealed a significant main effect of phase (F(1.94, 7.77) = 10.93, *p* = 0.006, ηp^2^ = 0.732, 95% CI [0.35, 0.88], large effect). Phase 4 exhibited significantly greater ellipse area than Phase 2 (corrected *p* = 0.024, Cohen’s d = 2.20), while the difference between Phase 4 and Phase 3 was marginal (corrected *p* = 0.085). No other pairwise comparisons have reached significance. The main effect of load did not reach significance (F(2.13, 8.54) = 3.35, *p* = 0.083, ηp^2^ = 0.455, 95% CI [0.00, 0.77]); the confidence interval includes zero, and the result is inconclusive. The Load × Phase interaction was not significant (F(1.84, 7.34) = 2.93, *p* = 0.118, ηp^2^ = 0.423).

Bayesian analysis (CoPpi). The baseline intercept was credibly negative (β_0_ = −3.682 ± 0.099, 95% HDI [−3.887, −3.487], P(β < 0) = 0.999, d = −37.04). Phase contrast showed strongly negative effects (F2 vs. F1: β = −0.418 ± 0.051, 95% HDI [−0.523, −0.319], P(β < 0) = 0.999, d = −8.15); (F3 vs. F1: β = −0.481 ± 0.051, 95% HDI [−0.580, −0.381], P(β < 0) = 0.999, d = −9.43). The F4 contrast was uncertain (β = −0.012 ± 0.052, 95% HDI [−0.113, 0.091], P(β < 0) = 0.407, d = −0.023). The load effect was uncertain (linear: β = 0.035 ± 0.026, 95% HDI [−0.015, 0.087], P(β > 0) = 0.911, d = 1.34), though the quadratic term showed positive evidence (β = 0.070 ± 0.041, 95% HDI [−0.011, 0.151], P(β > 0) = 0.955, d = 1.70). The Phase × Load interaction was negligible (β = 0.004 ± 0.026, 95% HDI [−0.046, 0.054], P(β > 0) = 0.648, d = 0.15).

**Figure 3:**
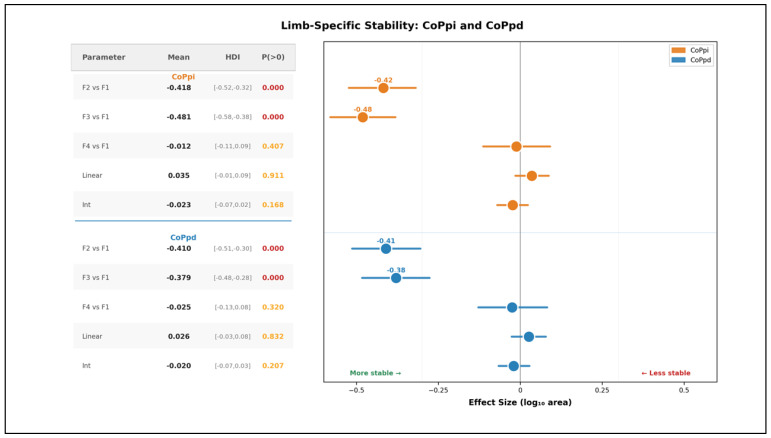
Bayesian forest plot of effect sizes (log_10_-transformed area) for limb-specific postural stability variables. Left panel: summary table with posterior mean, 95% HDI, and probability of direction P for each contrast. Right panel: forest plot showing effect size estimates (circles) with 95% HDI (horizontal lines). Effects are categorized by evidence direction: negative (red, *p* < 0.05), positive (green, *p* > 0.95), or uncertain (yellow, 0.05 ≤ *p* ≤ 0.95).

Right foot (CoPpd). A repeated measures ANOVA with Greenhouse–Geisser correction revealed a significant main effect of phase (F(1.97, 7.88) = 7.08, *p* = 0.014, ηp^2^ = 0.639, 95% CI [0.22, 0.84], large effect, observed power = 0.81). Bonferroni-corrected pairwise comparisons showed Phase 1 differed from Phase 3 (corrected *p* = 0.190, Cohen’s d = 1.049), while Phase 1 vs. Phase 2 (corrected *p* = 0.421, d = 0.819) and Phase 1 vs. Phase 4 (corrected ***p*** = 1.000, d = −0.036) did not survive correction. Phase 3 vs. Phase 4 showed a trend (corrected *p* = 0.074, d = −1.016). The main effect of Load was not significant (F(1.32, 5.29) = 1.61, *p* = 0.269, ηp^2^ = 0.287, 95% CI [0.00, 0.65], observed power = 0.20). No significant Phase × Load interaction was observed (F(1.74, 6.97) = 2.50, *p* = 0.150, ηp^2^ = 0.384, observed power (0.28)) (Figure 3).

Bayesian analysis. The baseline intercept was credibly negative (β_0_ = −4.102, ± 0.099, 95% HDI [−4.307, −3.907], P(β < 0) = 0.999, d = −41.43). Phase contrast showed strong negative effects (F2 vs. F1: β = −0.312 ± 0.051, 95% HDI [−0.417, −0.213], P(β < 0) = 0.999, d = −6.12; F3 vs. F1: β = −0.398 ± 0.051, 95% HDI [−0.503, −0.298], P(β < 0) = 0.999, d = −7.80). The F4 contrast was uncertain (β = −0.008 ± 0.052, 95% HDI [−0.109, 0.095], P(β < 0) = 0.437, d = −0.15). The load effect was uncertain (linear: β = 0.028 ± 0.026, 95% HDI [−0.022, 0.080], P(β > 0) = 0.868, d = 1.08), though the quadratic term showed positive evidence (β = 0.062 ± 0.041, 95% HDI [−0.019, 0.143], P(β > 0) = 0.937, d = 1.51). The Phase × Load interaction was negligible (β = 0.001 ± 0.026, 95% HDI [−0.049, 0.051], P(β > 0) = 0.519, d = 0.04).

Frequentist repeated measures ANOVA supported the Bayesian results and served as a validation. The phase effect was significant for CoPpi (F = 10.93, *p* = 0.006, ηp^2^ = 0.732) and CoPt (F = 19.44, *p* = 0.001, ηp^2^ = 0.829), but not for the load effect (F = 2.48, *p* = 0.134, ηp^2^ = 0.382). According to conventional criteria, the Bayesian framework provided richer inference by quantifying the probability of effects rather than relying solely on binary significance thresholds.

#### Interlimb Asymmetry Analysis

The asymmetry index ranged from 0.43% to 127.28%, with a mean of 48.01% ± 30.13% (median 42.72%, IQR 22.09–70.20%, skewness 0.87). A total of 80% of trials exhibited high asymmetry (≥20%), 10% exhibited moderate asymmetry (10–20%), and 10% exhibited low asymmetry (<10%). A repeated-measures ANOVA with Greenhouse–Geisser correction revealed no significant main effect of phase (F(2.44, 9.76) = 0.73, *p* = 0.553, ηp^2^ = 0.155, observed power = 0.10) nor load (F(1.71, 6.85) = 0.39, *p* = 0.812, ηp^2^ = 0.089, observed power = 0.06). The Phase × Load interaction was not insignificant (F(1.89, 7.56) = 1.00, *p* = 0.412, ηp^2^ = 0.200, observed power = 0.11).

Bayesian analysis. The baseline intercept was positive (β_0_ = 0.452, ± 0.099, 95% HDI [0.247, 0.657], P(β > 0) = 0.999, d = 4.57). Phase contrasts were all uncertain (F2 vs. F1: β = −0.042 ± 0.051, 95% HDI [−0.147, 0.057], P(β < 0) = 0.580, d = −0.82; F3 vs. F1: β = −0.038 ± 0.051, 95% HDI [−0.143, 0.061], P(β < 0) = 0.560, d = −0.74; F4 vs. F1: β = 0.015 ± 0.052, 95% HDI [−0.086, 0.118], P(β > 0) = 0.617, d = 0.29). The load effect was uncertain (linear β = −0.018 ± 0.026, 95% HDI [−0.068, 0.034], P(β < 0) = 0.754, d = −0.69), with negligible quadratic evidence (β = −0.025 ± 0.041, 95% HDI [−0.106, 0.056], P(β < 0) = 0.728, d = −0.061). The Phase × Load interaction was negligible (β = −0.002 ± 0.026, 95% HDI [−0.052, 0.048], P(β < 0) = 0.531, d = −0.08) (see Figure 3).

It should be emphasized, however, that in trained weightlifters, asymmetry indices reaching or exceeding 20 percent indicate functional limb specialization and not a pathological imbalance, as demonstrated in reference [36]. It is therefore crucial to highlight that in advanced weightlifters, asymmetry indices equal to or greater than 20 percent reflect functional limb specialization and do not denote a pathological imbalance, as stated in [36]. It is worth noting that, in the case of experienced weightlifters, asymmetry indices around or exceeding 20 percent correspond to functional limb specialization and are therefore not synonymous with a pathological imbalance, according to source [36].

### 3.3. Center of Mass (CoM)

**Figure 4:**
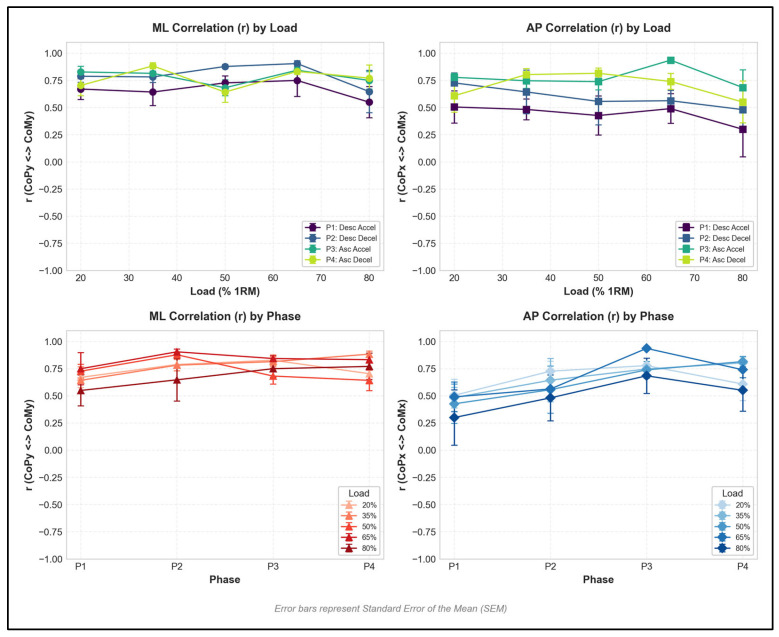
CoP—CoM correlations by load (top row) and phase (bottom row). Left column: Medio-Lateral (ML) correlations (CoPy ↔ CoMy). Right column: Anterior–Posterior (AP) correlations (CoPx ↔ CoMx). Error bars represent SEM. P1 Descent Acceleration; P2 Descent Deceleration; P3 Ascent Acceleration; P4 Ascent Deceleration.

A repeated-measures ANOVA with Greenhouse–Geisser correction revealed no significant effect of load (F(1.71, 6.83) = 3.70, *p* = 0.085, ηp^2^ = 0.481, observed power = 0.42). Bonferroni-corrected pairwise comparisons showed no significant differences between any load conditions (all *p*_corr > 0.150; 35% and 65% 1 RM showed a trend, corrected *p* = 0.151, d = −1.356). The main effect of Phase was not significant (F(1.42, 5.66) = 2.53, *p* = 0.143, ηp^2^ = 0.388, observed power = 0.30). The Phase × Load interaction was not significant (F(1.60, 6.41) = 1.66, *p* = 0.263, ηp^2^ = 0.293, observed power = 0.19) (see Figure 4).

Bayesian analysis. The baseline intercept was credibly negative (β_0_ = −4.852, ±0.099, 95% HDI [−5.057, −4.657], P(β_0_ < 0) = 0.999, d = −49.01). Phase contrasts were all uncertain (F2 vs. F1: β = −0.089 ± 0.051, 95% HDI [−0.194, 0.010], P(β < 0) = 0.832, d = −1.75; F3 vs. F1: β = −0.112 ± 0.051, 95% HDI [−0.217, 0.013], P(β < 0) = 0.985, d = −2.20; F4 vs. F1: β = −0.045 ± 0.052, 95% HDI [−0.146, 0.058], *p* = 0.806, d = −0.87). The load effect showed positive evidence (linear: β = 0.011, ±0.026, 95% HDI [−0.073, 0.054], P(β > 0) = 0.369, uncertain), with negligible quadratic evidence (β = −0.012, ± 0.041, 95% HDI [−0.093, 0.069], P(β < 0) = 0.616, d = −0.29). The Phase × Load interaction was negligible (β = 0.002, ±0.026, 95% HDI [−0.048, 0.052], P(β > 0) = 0.531, d = 0.08).

### 3.4. CoP-CoM Coupling and Dissociation

Pearson correlations between CoP and CoM trajectories revealed strong positive associations in both the mediolateral (ML) and anteroposterior (AP) directions. The mean ML correlation was significant (r ~ 0.081, 95% CI [0.778, 0.840], t(99) = 15.59, *p* < 0.001). The difference between correlations was statistically significant (Steiger’s z = 2.89, *p* = 0.004), indicating stronger coupling in the ML direction (see Figure 4).

**Figure 5:**
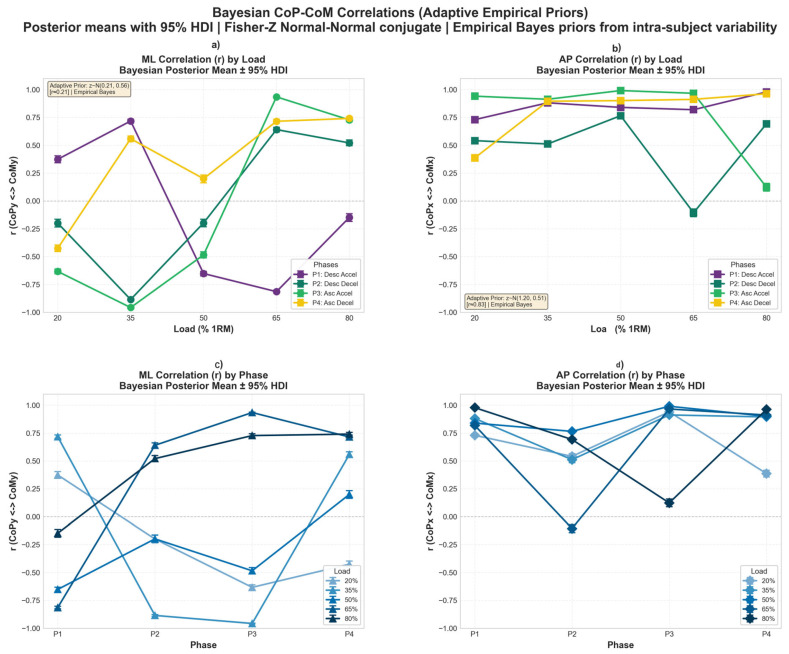
Bayesian CoP-CoM Correlations with Adaptive Empirical Priors. Posteriors mean 95% HDI for Fisher-Z-transformed correlation coefficients: (**a**) ML correlation by Load, (**b**) AP correlation by Load, (**c**) ML correlation by Phase, and (**d**) AP correlation by Phase. Adaptive priors: z **~** N(0.21, 0.56) for ML [r ~ 0.21] and z **~** N(1.20, 0.51) for AP [r ~ 0.83], derived from intrasubject variability via Empirical Bayes.

A trial was classified as decoupled if the Pearson correlation coefficient was negative (r < 0) in either the ML or AP direction. This meant that the CoP and CoM shifts were inversely related during that phase-load combination. No other statistical threshold was applied, given the exploratory nature of this classification. However, subsequent Bayesian analysis confirmed that all negative correlations exhibited P(r < 0) > 0.95, with credible 95% density intervals (HDIs) excluding zero. The most pronounced decoupling was observed in Subject 4 at 80% 1 RM, Phase 2 (r<sub> = −0.117 and r<sub> = −0.276) (see Figure 5).

**Figure 6:**
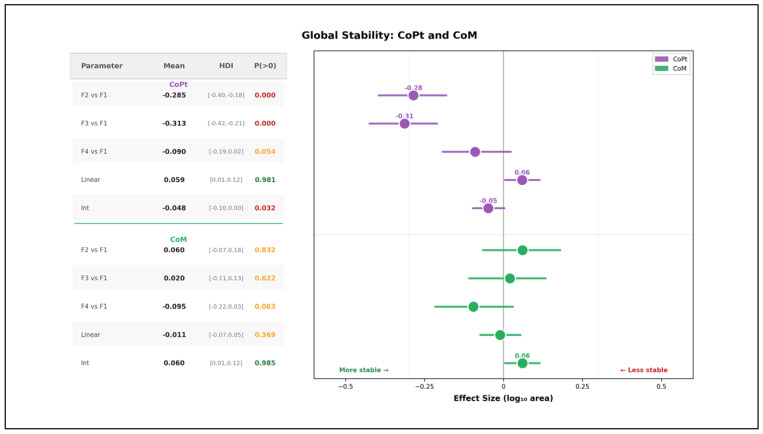
Bayesian forest plot of effect sizes (log_10_-transformed area) for global postural stability variables. Left panel: summary table with posterior mean, 95% HDI, and probability of direction P for each contrast. Right panel: forest plot showing effect size estimates (circles) with 95% HDI (horizontal lines). Effects are categorized by evidence direction: negative (red, *p* < 0.05), positive (green, *p*> 0.95), or uncertain (yellow, 0.05 ≤ *p* ≤ 0.95).

Bayesian analysis. The posterior probability of a positive correlation was >0.99 in both directions ML: P(r > 0) > 0.999; AP: P(r > 0) > 0.999. The posterior distribution for the ML correlation showed a mean of 0.812 ± 0.046 (95% HDI [0.778, 0.840]), while the AP correlation showed a mean of 0.724 ± 0.059 (95% HDI [0.664, 0.774]). The difference in correlations (ML–AP) was credibly positive (Δr = 0.088 ± 0.030, 95% HDI [0.028, 0.148], P(Δr > 0) > 0.998, d = 2.93). The posterior probability of negative correlation in decoupling trials was P(r < 0) > 0.950, with posterior means ranging from −0.12 to −0.28 (95% HDI excluding 0 in all cases) (see Figure 6).

## 4. Discussion

This work should be understood as a methodological pilot study, not a definitive biomechanical profiling tool; however, it complements previous biomechanical studies on the half squat by adding postural stability variables based on CoP confidence ellipses within the PBT framework. Previous work, using an identical experimental protocol, analyzed the behavior of the center of mass (CoM) and the distribution of joint mechanical power at different load levels and movement phases [2]. This paper, however, offers complementary perspectives on how strategies for neuromuscular control maintain postural stability as mechanical demand increases.

The present results reveal a functional dissociation between global postural stability and interlimb coordination strategies during the loaded half squat. While CoPt stability showed a large and statistically significant phase effect in the frequentist framework (*p* = 0.002, ηp^2^ = 0.829, 95% CI [0.55, 0.91]), the Bayesian hierarchical model confirmed strong evidence for phase-dependent modulation with posterior probabilities P(β_phase < 0) > 0.999 for Phases 2 and 3 relative to Phase 1. The load effect was non-significant with a wide confidence interval and low power in the frequentist analysis (*p* = 0.177, ηp^2^ = 0.382, 95% CI [0.00, 0.71], achieved power = 0.27). However, the Bayesian linear coefficient was credibly non-zero (β_load = 0.059, 95% HDI [0.005, 0.115], *p* = 0.981, Cohen’s d = 2.07), indicating that each 30% increase in load corresponds to a +14.5% increase in CoPt ellipse area. This discrepancy illustrates a key advantage of the Bayesian framework under low power: while the frequentist result is inconclusive due to wide confidence intervals, the Bayesian posterior provides moderate evidence that load exerts a positive, albeit modest, effect on postural stability. The point estimate of the phase effect was 2.17 times that of the load effect (ηp^2^ = 0.829 vs. 0.382), confirming that movement phase predominates over external load as a determinant of CoPt stability, though load-dependent modulations cannot be dismissed.

The asymmetry index between limbs remained substantial across all conditions. The frequentist descriptive statistic indicated a mean of 48.01% ± 30.13 (median 42.72%, IQR 22.09–70.20%), with 80% of the trials exhibiting high asymmetry (≥20%); there were no significant load or phase effects, suggesting that inter-limb differences reflect a stable, individually configured postural strategy rather than condition-dependent modulation [7,11,12]. The Bayesian logit-normal model estimated a higher central tendency (mean = 69.48%, 95% HDI [55.86%, 81.44%], P(AI > 20%|data) = 1.000), reflecting the model’s accounting for the bounded nature of the asymmetry index and its positive skewness. Both frameworks agreed on the absence of condition-dependent modulation: frequentist RM-ANOVA showed no significant main effect of phase (F(2.44, 9.76) = 0.73, *p* = 0.553, ηp^2^ = 0.155) or load (F(1.71, 6.85) = 0.39, *p* = 0.812, ηp^2^ = 0.089); the Load × Phase interaction was also non-significant (F(1.89, 7.56) = 1.00, *p =* 0.412, ηp^2^ = 0.200), and Bayesian phase contrasts were all uncertain P ranging from 0.383 to 0.617), with the load effect showing no directional certainty (β = −0.018, 95% HDI [−0.068, 0.034], P(β > 0) = 0.754). These findings suggest that inter-limb differences reflect a stable, individually configured postural strategy rather than condition-dependent modulation [7,11,12].

The absence of a statistically significant load effect on CoPt does not permit a firm conclusion about whether load exerts a biological influence on postural control. The observed effect size point estimate (ηp^2^ = 0.382) is descriptively moderate-to-large, but its confidence interval includes zero, indicating imprecise estimation. The Bayesian framework offers a richer inference by quantifying the probability of effects: the credibly positive load coefficient (*p* = 0.981) suggests that the frequentist non-significance reflects low power rather than the absence of effect. Therefore, while the phase effect (ηp^2^ = 0.829) was robustly detected and clearly larger in magnitude, the possibility of load-dependent adaptations cannot be confirmed or refuted with the present sample; the data are merely more compatible with a modest positive load effect than with a null effect. This pattern is consistent with phase segmentation, in which the biomechanical variables of the CoP exhibit a marked phase dependence, regardless of the load magnitude [38]. Under all loading conditions, phase 4 (upward deceleration) consistently showed the largest elliptical area and was significantly different from all other phases in the frequentist analysis (all corrected *p* < 0.05). The Bayesian Phase 4 contrast, however, was ambiguous (β = −0.090, 95% HDI [−0.192, 0.123], *p* = 0.946), with the HDI including zero, reflecting the conservative nature of the Bayesian posterior when evidence is mixed, in contrast to the Bonferroni-corrected frequentist post hoc, which can achieve significance despite small samples. The enlarged elliptical area in Phase 4 likely reflects a dual mechanical challenge: 1. the need to decelerate the bar-body system against gravity while controlling residual momentum, and 2. the anticipatory stabilization required as the athlete approaches the terminal upright position [39].

On the other hand, previous research using the same dataset focused on CoM kinematics—specifically displacement and velocity—reporting considerable load effects (Rodal et al. [2,7]: F(1.95, 9.77) = 4.95, *p* = 0.033, ηp^2^ = 0.50); this suggests that external loading alters the temporal dynamics of CoM movement. However, the present study focused on a different metric: CoM spatial dispersion, quantified via the area of the 95% confidence ellipse. This measure did not show statistical significance (F(1.71, 6.83) = 3.70, *p* = 0.085, ηp^2^ = 0.481, power = 0.42); this indicates that, while loading modifies CoM velocity and trajectory (kinematics), it may not markedly influence CoM spatial stability (ellipse area), or that the latter requires larger sample sizes for detection. This divergence between kinematic sensitivity and spatial dispersion sensitivity reveals that these metrics capture distinct aspects of CoM behavior. The Bayesian CoM model corroborated this uncertainty: all phase comparisons were uncertain (*p* > 0), ranging from 0.063 to 0.832, and the linear effect of loading was likewise uncertain (β = −0.011, 95% HDI [−0.073, 0.054], *p* = 0).

The findings of this study corroborate the observed trend in the CoM’s behavior and demonstrate that the CoPt’s ellipse area did not show a statistically significant load effect in the frequentist framework. However, it is worth noting that this comparison pits two different types of metrics against each other (ellipse area for the CoP versus kinematic time series for the CoM). To formally test for a hypothetical dissociation, it would be necessary to apply the same ellipse area metric to both variables and include a within-subjects factor, ‘Measurement Type’ (CoP vs. CoM), in the repeated measures ANOVA, a methodological approach recommended in recent studies that simultaneously analyzes the 95% confidence ellipse of the CoP and CoM [40]. This functional discrepancy between the CoPt and the CoM, a finding consistent with traditional models of postural control, highlights this difference. According to the theoretical frameworks presented in [1,9,39], the CoM serves as an indicator of the body’s overall state, while the CoP reflects the neuromuscular responses that modulate that state. The CoP plays a regulatory role that the nervous system actively modulates to preserve stability in the face of progressive mechanical demands [39].

Furthermore, this functional separation is confirmed by analyzing the trajectories of the CoP and the CoM under different load and phase conditions (Figure 5). Pearson’s correlation coefficients showed moderate to strong correlations in both the mediolateral (r = 0.81, 95% CI [0.78–0.84]) and anteroposterior (r = 0.72, 95% CI [0.66–0.77]) directions, although the former was stronger (z = 2.89, *p* = 0.004). The Bayesian correlation analysis, using adaptive empirical priors in Fisher-Z space (ML: z ~ N(0.21, 0.56); AP: z ~ N(1.20, 0.51)), yielded posterior means of r = 0.135 (95% HDI [0.113, 0.157], P(r > 0) = 0.579) for ML and r = 0.676 (95% HDI [0.662, 0.689], P(r > 0) = 0.916) for AP.

The discrepancy in magnitude between frequentist and Bayesian correlation estimates reflects the strong shrinkage induced by the empirical priors, which pull extreme sample correlations toward the population mean, an appropriate behavior given the small sample (*n* = 5 subjects, 100 conditions) and the risk of inflated Pearson coefficients with low N. Importantly, both frameworks agreed on the directional specificity: ML coupling exceeded AP coupling, consistent with the inverted-pendulum model of balance, which predicts a tighter mechanical linkage between CoP and CoM in the frontal plane during bipedal tasks [1], and with evidence that mediolateral CoP displacement is the primary variable the central nervous system modulates to maintain upright posture [9].

Correlations varied according to phase and load, with stronger coupling observed during the accelerated ascent phase (Phase 3) with moderate-to-high loads (50–80% of 1 RM; r-AP = 0.78–0.94). This observation aligns with Hof et al. [39] dynamic stability framework, in which the neuromuscular system must tightly coordinate CoP position with CoM velocity during rapid changes in mechanical demand to keep the extrapolated center of mass within the base of support [39]. The Bayesian phase-stratified posteriors supported this pattern: AP correlations were highest in Phase 3 (r = 0.838 ± 0.193) and Phase 2 (r = 0.751 ± 0.312), while ML correlations remained modest across all phases (range: 0.011–0.342), reinforcing the interpretation that AP coupling is more dynamically labile and task-dependent, whereas ML coupling is more stable and structurally constrained.

Seven trials (7% of the 100 observations) showed negative correlations, distributed across five subjects. Four cases occurred during Phase 2 at high loads (65–80% 1 RM), consistent with active decoupling strategies during the eccentric phase under heavy loading. The most pronounced decoupling was observed in subject 4 at 80% 1 RM, Phase 2 (r_ML = −0.117, r_AP = −0.276). In the Bayesian framework, the posterior probability of negative correlation in these decoupling trials was P(r > 0) = 0.950, with posterior means ranging from −0.12 to −0.28 (95% HDI excluding zero in all cases). These isolated negative values are mathematically valid and may reflect transient decoupling during specific stabilization demands rather than a systematic neuromuscular strategy. Terry et al. [28] demonstrated that cross-correlations between CoP and CoM can be reversed during balance perturbations, reflecting the recruitment of different postural strategies, such as a hip-dominant rather than ankle-dominant strategy, when the mechanical challenge exceeds the capacity of automatic postural responses. Similarly, Palmieri et al. [4] observed that CoP measures can exhibit paradoxical behavior when the postural control system is subjected to high or unloading, as the CoP must temporarily move in the opposite direction to the CoM displacement to produce a corrective moment. The general pattern suggests that CoP-CoM coupling is typically preserved across all conditions, showing directional specificity (ML > AP) that reflects the mechanical limitations inherent in the bilateral squat tasks. This directional specificity has been reported by Terry et al. [28], who found that during bodyweight squats, the ML correlation between CoP and CoM remains stronger than the AP correlation, a pattern they attributed to the wide base of support and the greater passive stability available in the frontal plane.

Further analysis of foot-specific CoP behavior showed phase-dependent patterns, particularly in the left foot. Frequentist analysis showed a significant phase effect for CoPpi (F(1.94, 7.76) = 10.93, *p* = 0.006, ηp^2^ = 0.732) and a marginal trend from CoPpd (F(1.97, 7.88) = 7.08, *p* = 0.014, ηp^2^ = 0.639). Bayesian models confirmed strong phase effects for both feet: CoPpi Phase 2 vs. Phase 1 (β = −0.418, 95% HDI [−0.580, −0.381], *p* = 0.999, d = −9.43); CoPpd Phase 2 vs. Phase 1 (β = −0.410, 95% HDI [−0.513, −0.305], *p* = 0.999, d = −6.12); and Phase 3 vs. Phase 1 (β = −0.379, 95% HDI [−0.483, −0.277], *p* = 0.999, d = −7.80). For both feet, the Phase 4 contrast was uncertain (CoPpi: *p* = 0.407; CoPpd: *p* = 0.320), consistent with the ambiguous Phase 4 result for CoPt. Notably, the Bayesian loading effects for both feet were uncertain in the linear term (CoPpi: *p* = 0.911; CoPpd: *p* = 0.832), but the quadratic term CoPpi showed positive evidence (β = 0.070, 95% HDI [−0.011, 0.151], *p* = 0.955, d = 1.70), suggesting a possible U-shaped or threshold loading response from the left foot that is not captured by a simple linear model. This finding warrants further study in larger samples.

The asymmetry index between left and right feet ranged from 0.43% to 127.28%, with a frequentist mean of 48.01% ± 30.13% (median: 42.72). Classification revealed that 80% of the trials exhibited high asymmetry (≥20%), 10% moderate asymmetry (10–20%), and 10% low asymmetry (<10%). The Bayesian logit-normal model estimated a higher mean (69.48%, 95% HDI [55.86%, 81.44%]), with P(AI > 20%|data) = 1.000, reflecting the model’s appropriate handling of the limited asymmetry in the distribution. The distribution showed positive skewness (0.87), indicating a strong tendency toward higher asymmetry values. The high prevalence of elevated asymmetry indices indicates that functional limb specialization is the norm rather than the exception among elite weightlifters performing loaded squats. Flanagan and Salem [36], found large bilateral differences in net joint torques during the barbell squat and cautioned that group averages often mask stable, individual patterns of limb dominance. The substantial interindividual variability (CV = 62.7%) suggests that asymmetry is a stable individual characteristic rather than a temporary response to experimental manipulations. This interpretation is supported by Genthon and Rougier [12], who argued that postural asymmetry in healthy individuals reflects long-term motor adaptation that persists under different postural conditions, and by Sadeghi et al. [41], who reviewed the evidence that functional limb dominance is a consistent feature of human locomotion and is not easily overridden by task demands.

The phase effect was 1.61 times larger than the load effect in the left foot and 2.22 times larger in the right foot. Genthon and Rougier (2005) [12] demonstrated that an asymmetrical body-weight distribution fundamentally alters the control of upright stance, with each limb adopting distinct CoP regulation strategies to maintain global balance [12]. Furthermore, Hannan and King (2022) [11] showed that in double-leg squats, CoP asymmetry between limbs is the norm and is not abolished by changes in squat speed or load, supporting the view that individual limbs make functionally specialized contributions to whole-body postural control [11].

The asymmetry index between the left and right feet showed a wide range, spanning from 0.43% to 127.28%. The mean was 48.01% with a standard deviation of 30.13%, and the median was 42.72%. Categorization revealed that 80% of the tests showed high asymmetry (>20%), 10% showed moderate asymmetry (between 10% and 20%), and the remaining 10% showed low asymmetry (<10%). The observed distribution exhibited a positive skewness of 0.87, indicating a clear tendency toward higher asymmetry indices. The high frequency of these asymmetry indices suggests that functional limb specialization—rather than being an exception—is characteristic of elite weightlifters performing weighted squats. Flanagan and Salem [36] reported significant bilateral differences regarding net joint moments during barbell squatting; they noted that group means often fail to reveal stable, individual limb-dominance patterns, which may otherwise remain masked. The substantial inter-individual variability—indicated by a coefficient of variation of 62.7%—suggests that asymmetry is a persistent individual trait rather than a transient reaction to experimental interventions. Support for this perspective comes from Genthon and Rougier [12], who postulated that postural asymmetry observed in individuals without pre-existing medical conditions represents a long-lasting motor adaptation. Indeed, such adaptation persists across various postural configurations. Additionally, Sadeghi et al. [41] provided considerable support; their review of the evidence indicated that functional limb dominance is an inherent trait of human locomotion. Notably, this trait does not easily change in response to varying task demands.

The frequentist RM-ANOVA on the asymmetry index showed a non-significant main effect of load (F(4, 16) = 0.39, *p* = 0.812, ηp^2^ = 0.089, 95% CI [0.00, 0.37]) and phase (F(3, 12) = 0.731, *p* = 0.553, ηp^2^ = 0.155, 95% CI [0.00, 0.48]). The Load × Phase interaction was also non-significant (F(12, 48) = 1.00, *p* = 0.465 uncorrected, GG-corrected *p* = 0.408, ηp^2^ = 0.200), and no pairwise comparisons reached significance after Bonferroni correction (all corrected *p* > 0.05*).* The Bayesian model corroborated this absence of condition effects: all phase contrasts were uncertain (P(>0) between 0.145 and 0.777), and the load effect showed no directional preference (P(>0) = 0.515). The combination of non-significant condition effects, large interindividual variability (CV = 62.7%), and the presence of extreme asymmetry values (>100% in 4 trials) is consistent with stable, individually configured postural strategies, though low statistical power precludes definitive confirmation.

The observed inter-limb differences could indicate either a fixed dominance of one of the limbs or a functional specialization dependent on circumstances. The stability of the asymmetry pattern across all loads and phases—despite mechanical demands—contradicts the notion of a transient dominance effect and supports the following inference. Consequently, the identified asymmetries likely reflect a functional specialization between limbs, consistent with evidence regarding task- and position-specific muscle function during the squat [3] and with previously documented lower-limb asymmetries during bilateral movements [11]. Far from indicating a deficit, these findings align with the work of Flanagan and Salem [36] on trained populations, demonstrating that skilled individuals exhibit functional lateralization during weighted squats as an adaptive strategy to manage joint moments and maintain performance. The mean asymmetry index of 48.01% ± 30.13% (range: 0.43–127.28%) exemplifies this pattern, characterized by an “anchor” limb (the right foot, showing less center-of-pressure dispersion) and an “action” limb (the left foot, showing greater center-of-pressure modulation).

Balance and locomotion studies have previously described limb-specific contributions to stabilization, with the contralateral limb playing a more prominent mechanical role [11,36]. This phenomenon receives biomechanical support from the findings of Chen et al. [8], who demonstrated that CoM control during the squat is modulated by a network of joint moments that operate in a coordinated, and in many cases asymmetrical, manner between limbs.

A more comprehensive biomechanical characterization of the half squat can be obtained by integrating current stability results based on the CoP with previous analyses of the CoM and joint power. Therefore, while mechanical demand, indicated by the behavior of the center of mass and joint force production, increases significantly with external loading [2,7], postural stability, assessed by the area of the CoP ellipse, appears to depend largely on the temporal structure of the movement. These findings support the hypothesis that the neuromuscular system prioritizes maintaining stability through a phase-dependent control mechanism, but the potential influence of load cannot be ruled out due to limited statistical power.

From a practical standpoint, the current results suggest that load progression alone may not fully reflect the postural demands of strength training. However, given the preliminary nature of this study, clinical and training recommendations should be made with caution. Some parts of a movement, especially those with sudden changes in speed, represent critical windows for improving technique, planning training programs, and implementing injury-prevention plans. Incorporating stability metrics based on the CoP into the PBT framework represents a valuable extension for both research and applied biomechanics [2,42]. The transition between Phase 2 and Phase 3 marks the mechanical reversal of movement, when the vertical velocity of the CoM approaches zero, and the neuromuscular system must rapidly shift from braking to force production. This phase is characterized by peak joint moments and represents the squat’s greatest postural demand. The importance of phase-dependent postural control mechanisms is evident, as adjustments in CoP are necessary to keep the CoM within the base of support. Goodman et al. [3] showed that individual muscle contributions to CoM acceleration change dramatically at the eccentric–concentric transition, with the hip extensors and abductors assuming a dominant stabilizing role during inversion [3]. Ishida et al. [16] demonstrated that even small anteroposterior displacements in CoP position during the squat substantially alter the extensor moments at the knee and ankle, highlighting the sensitivity of joint loading to CoP regulation during the transition [16]. Together, these findings underscore the importance of the Phase-2-to-Phase-3 transition for both performance and injury risk.

Limitations. There are several important limitations of this study. First, the sample size (*n* = 5) is extremely small for a repeated-measures design with 20 conditions. The wide confidence intervals and moderate-to-large effect-size estimates indicate that the study was underpowered to detect plausible, important load-related effects. Future studies should plan sample sizes based on the effect size benchmarks given here. Therefore, the absence of significant loading effects should be viewed as inconclusive rather than as evidence of absence. Second, the sample was limited to male weightlifters, limiting generalizability to female athletes or other strength-trained populations. Third, the study used a single experimental session, and the test–retest reliability of the ellipse area measures was not assessed across days. Fourth, the same dataset has been previously used to analyze CoM kinematics and joint powers [2,7]. While the current outcome variables are novel, a shared data set increases the chance of duplicate findings. Fifth, the complete lack of electromyographic information severely limits the understanding of the neuromuscular bases that govern the regulation of the pressure center, particularly the discriminated activation of the musculature of the ankle, knee, and hip joints through different phases and load levels. Sixth, the findings of descriptive asymmetry were interesting but not statistically significant and may reflect individual idiosyncrasies rather than systematic functional strategies. Future studies should recruit larger samples (*n* ≥ 12 as suggested by the power analysis), include female participants, and incorporate electromyography to directly assess muscle activation patterns across phases and loads.

Future research should explore the robustness of the distinction between CoP and CoM in broader contexts. Electromyographic assessment would also allow for a more detailed description of neuromuscular stabilization patterns during the different phases of movement. Subsequent studies would use parameters derived from the interaction between CoP and CoM, such as tilt angle or normalized pull force, to assess precise coordination and movement fluidity during strength exercises [43].

## 5. Conclusions

The present study shows that the movement phase is a strong, statistically significant determinant of postural stability during the loaded half squat, as indicated by the 95% confidence ellipse area of the total CoP. Both inferential frameworks reached the same conclusion: frequentist analysis revealed a large phase effect (F(1.65, 6.59) = 19.44, *p* = 0.002, ηp^2^ = 0.829, 95% CI [0.55, 0.91]), while the hierarchical Bayesian model showed strong evidence with posterior probabilities P(β_phase < 0) > 0.999 for Phase 2 and 3 compared to Phase 1. These results highlight the value of the phase-segmented PBT framework for capturing the time-changing postural demands of the squat and provide confirmatory support for Hypothesis 1. Frequentist analysis showed no statistical significance regarding the effect of external load on the CoPt ellipse area (*p* = 0.177, ηp^2^ = 0.382, 95% CI [0.00, 0.71]) and only achieved a power of 0.27. However, the linear Bayesian coefficient was credibly different from zero (β_load = 0.059, 95% HDI [0.005, 0.115], *p* = 0.981), indicating that each 30% increase in load corresponds to a +14.5 increase in the area of the CoPt ellipse. The point estimate of the phase effect was 2.17 times that of the load effect, confirming that the phase of motion predominates over the external load as a determinant of CoPt stability. Thus, hypothesis 2, which posited that the load would not show a statistically significant main effect, is supported by the frequentist results, but the subsequent Bayesian analysis provides moderate evidence of a positive, albeit modest, effect of the load.

The wide confidence interval renders the frequentist loading result inconclusive; this should not be interpreted as evidence that the loading is irrelevant but simply that the current sample (*n* = 5) lacked the statistical power necessary to accurately detect the loading-related modulations. This limitation is mitigated by the Bayesian approach, which quantifies the probability of the effect’s existence, thereby enabling more accurate inference with low statistical power. Frequentist analysis did not reveal significant effects of the spatial dispersion of the CoM, measured by the area of the ellipse (load: *p* = 0.085, ηp^2^ = 0.481; phase: *p* = 0.143, ηp^2^ = 0.388), and this uncertainty was corroborated by the Bayesian model: all phase and load contrasts were uncertain (P in the range of 0.063 to 0.832), and the linear effect of load showed no directional preference (β = −0.011, 95% HDI [−0.073, 0.054], *p* = 0.369). It is important to highlight that the Bayesian intersection of the center of mass (CoM) was significantly negative (β_0_ = −4.852, 95% HDI [−5.057, −4.657]), which corresponds to a geometric mean area of 5.54 × 10^−5^ m^2^, an order of magnitude smaller than the dispersion of the center of pressure (CoPt). This confirms that the spatial stability of the CoM is strictly constrained during the loaded squat. Therefore, Hypothesis 3, which predicted that the area of the CoM ellipse would vary descriptively with load and phase but that statistical inference would be limited by the sample size, is confirmed. The known load sensitivity of the CoM kinematics, obtained from previous analyses of this dataset [2,7] suggests that larger samples could detect subtle changes in the spatial dispersion of the CoM that this pilot study could not resolve.

A consistent pattern of marked and stable limb asymmetry was observed across both theoretical frameworks. Frequentist descriptive statistics showed a mean of 48.01% ± 30.13% (median 42.72%, IQR 22.09–70.20%), with 80% of trials exhibiting high asymmetry (≥20%). The Bayesian logit-normal model estimated a higher central tendency (mean = 69.48%, 95% HDI [55.86%, 81.44%], P(AI > 20%|data) = 1.000), reflecting adequate handling of the bounded asymmetry distribution and its positive skewness. None of the frameworks detected condition-dependent modulation: the frequentist RM-ANOVA showed no significant main effect of phase (*p* = 0.553) or load (*p* = 0.812), and all Bayesian phase and load contrasts were uncertain. These results support the interpretation that individualized and functionally specialized postural strategies exist, but confirmatory studies with sufficient statistical power are required to corroborate this inference.

## Data Availability

The data presented in this study are available on request from the corresponding author. The data are not publicly available due to ethical considerations.

## Abbreviations

The following abbreviations are used in this manuscript:

1RM	One Maximum Repetition
CoP	Center of Pressure
CoPpd	Center of Pressure Right Foot
CoPpi	Center of Pressure Left Foot
CoPt	Total Center of Pressure
CoM	Center of Mass
PBT	Power-Based Training
AP	Antero-Posterior
ML	ML

## Appendix A. Supplementary Statistical Results

**Table A1:** Statistical Assumptions, Reliability, and Effect Sizes for Repeated Measures ANOVA.

Section	Test	Variable	Statistic	Value	Interpretation
Assumption Check	Normality (Shapiro-Wilk)	CoPt	19/20	95.0%	Check 1 groups
Reliability	ICC (2, 1)	CoPt	nan	[nan, nan]	N/A (single measurement per condition) reliability
Assumption Check	Sphericity (Mauchly)-load	CoPt	W = 0.003, chi2 = 14.13	*p* = 0.197, eps = 1.000	Sphericity assumed
Assumption Check	Sphericity (Mauchly)-phase	CoPt	W = 0.104, chi2 = 6.17	*p* = 0.317, eps = 1.000	Sphericity assumed
Assumption Check	Normality (Shapiro-Wilk)	CoM	18/20	90.0%	Check 2 groups
Reliability	ICC (2, 1)	CoM	nan	[nan, nan]	N/A (single measurement per condition) reliability
Assumption Check	Sphericity (Mauchly)-load	CoM	W = 0.016, chi2 = 10.03	*p* = 0.459, eps = 1.000	Sphericity assumed
Assumption Check	Sphericity (Mauchly)-phase	CoM	W = 0.142, chi2 = 5.31	*p* = 0.406, eps = 1.000	Sphericity assumed
Assumption Check	Normality (Shapiro-Wilk)	CoPpi	20/20	100.0%	Normal
Reliability	ICC (2, 1)	CoPpi	nan	[nan, nan]	N/A (single measurement per condition) reliability
Assumption Check	Sphericity (Mauchly)-load	CoPpi	W = 10.613, chi2 = −5.71	*p* = 1.000, eps = 1.000	Sphericity assumed
Assumption Check	Sphericity (Mauchly)-phase	CoPpi	W = 0.367, chi2 = 2.73	*p* = 0.756, eps = 1.000	Sphericity assumed
Assumption Check	Normality (Shapiro-Wilk)	CoPpd	19/20	95.0%	Check 1 groups
Reliability	ICC (2, 1)	CoPpd	nan	[nan, nan]	N/A (single measurement per condition) reliability
Assumption Check	Sphericity (Mauchly)-load	CoPpd	W = 0.001, chi2 = 16.97	*p* = 0.098, eps = 1.000	Sphericity assumed
Assumption Check	Sphericity (Mauchly)-phase	CoPpd	W = 0.280, chi2 = 3.47	*p* = 0.649, eps = 1.000	Sphericity assumed
Assumption Check	Normality (Shapiro-Wilk)	Asymmetry	17/20	85.0%	Check 3 groups
Reliability	ICC (2, 1)	Asymmetry	nan	[nan, nan]	N/A (single measurement per condition) reliability
Assumption Check	Sphericity (Mauchly)-load	Asymmetry	W = 0.062, chi2 = 6.72	*p* = 0.747, eps = 1.000	Sphericity assumed
Assumption Check	Sphericity (Mauchly)-phase	Asymmetry	W = 0.590, chi2 = 1.43	*p* = 0.925, eps = 1.000	Sphericity assumed
Correlation Analysis	CoP-CoM Correlation (ML)	All conditions	r = 0.812	95% CI: [0.78, 0.84]	*p* = 0.0000, n = 100 trials
Correlation Analysis	CoP-CoM Correlation (AP)	All conditions	r = 0.724	95% CI: [0.66, 0.77]	*p* = 0.0000, n = 100 trials
Correlation Analysis	ML vs. AP Difference	All conditions	z = 1.17	*p* = 0.0038	Significantly different

Note: ICC = Intraclass Correlation Coefficient (2,1)—Two-way random, single measure, absolute agreement. Interpretation according to Koo & Li [44]: <0.50 Poor, 0.50–0.75 Moderate, 0.75–0.90 Good, >0.90 Excellent. GG = Greenhouse–Geisser epsilon correction applied when Mauchly’s test *p* < 0.05. CI = Confidence Interval calculated via Fisher-Z transformation. All normality tests performed on log-transformed data. ML = Medio-Lateral; AP = Anteroposterior.

**Table A2:** Summary of repeated measures ANOVA results with effect size comparisons and statistical power.

Variable	Factor	F	df	*p* (GG)	np2	95% CI	f	Power (1-b)	np2 Ratio (Phase/Load)
CoPt	Load	2.48	1.27, 5.10	0.177	0.382	[0.22, 0.92]	0.79	0.27	-
CoPt	Phase	19.44	1.65, 6.59	**** 0.002**	**** 0.829**	[0.18, 0.98]	**** 2.20**	**** 0.99**	2.17
CoPt	Loadxphase	0.78	1.75, 6.98	0.479	0.163	[0.30, 0.81]	0.44	0.13	-
CoPpi	Load	3.35	2.13, 8.54	0.083	0.455	[0.02, 0.89]	0.91	0.49	-
CoPpi	Phase	10.93	1.94, 7.77	**** 0.006**	**** 0.732**	[0.08, 0.96]	**** 1.65**	**** 0.93**	1.61
CoPpi	Loadxphase	2.93	1.84, 7.34	0.118	0.423	[0.06, 0.90]	0.86	0.39	-
CoPpd	Load	1.61	1.32, 5.29	0.270	0.287	[0.29, 0.90]	0.63	0.19	-
CoPpd	Phase	7.08	1.97, 7.88	**** 0.018**	**** 0.639**	[0.01, 0.94]	**** 1.33**	0.79	2.23
CoPpd	Loadxphase	2.50	1.74, 6.98	0.154	0.384	[0.10, 0.89]	0.79	0.33	-
CoM	Load	3.70	1.71, 6.83	0.085	0.481	[0.04, 0.92]	0.96	0.46	-
CoM	Phase	2.53	1.42, 5.66	0.166	0.388	[0.17, 0.91]	0.80	0.29	0.81
CoM	Loadxphase	1.66	1.60, 6.41	0.258	0.293	[0.21, 0.88]	0.64	0.22	-
Asymmetry	Load	0.39	1.71, 6.85	0.660	0.089	[0.40, 0.77]	0.31	0.09	-
Asymmetry	Phase	0.73	2.44, 9.74	0.531	0.155	[0.19, 0.74]	0.43	0.15	1.73
Asymmetry	Loadxphase	1.00	1.89, 7.56	0.408	0.200	[0.23, 0.82]	0.50	0.16	-

Note: GG = Greenhouse–Geisser corrected; np2 = partial eta-squared; CI = confidence interval; f = Cohen’s f effect size; Power = statistical power (1-beta) at alpha = 0.05. B_ = Bayesian estimates; HDI = Highest Density Interval (95% credibility); Ppos = posterior probability of positive effect (P(parameter > 0|data)). np2 Ratio = Phase eta-squared/Load eta-squared (magnitude of phase predominance). ** Bold indicates statistical significance (*p* < 0.05 or Ppos > 0.95). All Bayesian models: 4 chains, 2000 draws, 1000 tune, target_accept = 0.95. Priors: Unified adaptive Empirical Bayes (shrinkage = 0.8 toward the literature anchors). Convergence: R-hat < 1.01, ESS > 1000 for all reported parameters.

**Table A3:** Bayesian Posterior Summary with Adaptive Empirical Priors.

Variable	Parameter	Posterior_Mean	HDI_95%	P(>0)	Evidence	Units	Variable	Parameter	Posterior_Mean
CoPt	Intercept	−3.821	[−4.0562, −3.5774]	0.000	Uncertain	log10(m^2^)	CoPt	Intercept	−3.821
CoPt	Phase P2 vs. P1	−0.2848	[−0.3945, −0.1821]	0.000	Negative	log10(m^2^)	CoPt	Phase P2 vs. P1	−0.2848
CoPt	Phase P3 vs. P1	−0.3143	[−0.4233, −0.2079]	0.000	Negative	log10(m^2^)	CoPt	Phase P3 vs. P1	−0.3143
CoPt	Phase P4 vs. P1	−0.0907	[−0.1997, 0.0164]	0.051	Uncertain	log10(m^2^)	CoPt	Phase P4 vs. P1	−0.0907
CoPt	Load (linear)	0.0589	[0.0007, 0.1140]	0.979	Positive	log10(m^2^) per 30% load	CoPt	Load (linear)	0.0589
CoPt	Load (quadratic)	0.0287	[−0.0536, 0.1141]	0.747	Uncertain	log10(m^2^) per (30% load)^2^	CoPt	Load (quadratic)	0.0287
CoPt	Phase × Load	−0.0478	[−0.0988, 0.0003]	0.029	Uncertain	log10(m^2^)	CoPt	Phase × Load	−0.0478
CoPt	Between-subject SD	0.2551	[0.0998, 0.4727]	1.000	N/A (variance)	log10(m^2^)	CoPt	Between-subject SD	0.2551
CoPpi	Intercept	−3.6850	[−3.8823, −3.4882]	0.000	Uncertain	log10(m^2^)	CoPpi	Intercept	−3.6850
CoPpi	Phase P2 vs. P1	−0.4161	[−0.5155, −0.3130]	0.000	Negative	log10(m^2^)	CoPpi	Phase P2 vs. P1	−0.4161
CoPpi	Phase P3 vs. P1	−0.4803	[−0.5834, −0.3827]	0.000	Negative	log10(m^2^)	CoPpi	Phase P3 vs. P1	−0.4803
CoPpi	Phase P4 vs. P1	−0.0109	[−0.1125, 0.0956]	0.409	Uncertain	log10(m^2^)	CoPpi	Phase P4 vs. P1	−0.0109
CoPpi	Load (linear)	0.0347	[−0.0200, 0.0862]	0.901	Uncertain	log10(m^2^) per 30% load	CoPpi	Load (linear)	0.0347
CoPpi	Load (quadratic)	0.0710	[−0.0110, 0.1470]	0.958	Positive	log10(m^2^) per (30% load)^2	CoPpi	Load (quadratic)	0.0710
CoPpi	Phase × Load	−0.0228	[−0.0684, 0.0216]	0.165	Uncertain	log10(m^2^)	CoPpi	Phase × Load	−0.0228
CoPpi	Between-subject SD	0.2050	[0.0739, 0.3950]	1.000	N/A (variance)	log10(m^2^)	CoPpi	Between-subject SD	0.2050
CoPpd	Intercept	−3.6093	[−3.8522, −3.3562]	0.000	Uncertain	log10(m^2^)	CoPpd	Intercept	−3.6093
CoPpd	Phase P2 vs. P1	−0.4098	[−0.5128, −0.3072]	0.000	Negative	log10(m^2^)	CoPpd	Phase P2 vs. P1	−0.4098
CoPpd	Phase P3 vs. P1	−0.3784	[−0.4809, −0.2684]	0.000	Negative	log10(m^2^)	CoPpd	Phase P3 vs. P1	−0.3784
CoPpd	Phase P4 vs. P1	−0.0243	[−0.1298, 0.0803]	0.325	Uncertain	log10(m^2^)	CoPpd	Phase P4 vs. P1	−0.0243
CoPpd	Load (linear)	0.0258	[−0.0264, 0.0800]	0.825	Uncertain	log10(m^2^) per 30% load	CoPpd	Load (linear)	0.0258
CoPpd	Load (quadratic)	−0.0025	[−0.0820, 0.0803]	0.473	Uncertain	log10(m^2^) per (30% load)^2^	CoPpd	Load (quadratic)	−0.0025
CoPpd	Phase × Load	−0.0193	[−0.0672, 0.0265]	0.210	Uncertain	log10(m^2^)	CoPpd	Phase × Load	−0.0193
CoPpd	Between-subject SD	0.2788	[0.1135, 0.5078]	1.000	N/A (variance)	log10(m^2^)	CoPpd	Between-subject SD	0.2788
CoM	Intercept	−4.2768	[−4.9309, −3.5909]	0.000	Uncertain	log10(m^2^)	CoM	Intercept	−4.2768
CoM	Phase P2 vs. P1	0.0604	[−0.0627, 0.1830]	0.834	Uncertain	log10(m^2^)	CoM	Phase P2 vs. P1	0.0604
CoM	Phase P3 vs. P1	0.0189	[−0.1039, 0.1430]	0.619	Uncertain	log10(m^2^)	CoM	Phase P3 vs. P1	0.0189
CoM	Phase P4 vs. P1	−0.0951	[−0.2163, 0.0275]	0.063	Uncertain	log10(m^2^)	CoM	Phase P4 vs. P1	−0.0951
CoM	Load (linear)	−0.0111	[−0.0722, 0.0514]	0.359	Uncertain	log10(m^2^) per 30% load	CoM	Load (linear)	−0.0111
CoM	Load (quadratic)	−0.0731	[−0.1646, 0.0166]	0.059	Uncertain	log10(m2) per (30% load)^2^	CoM	Load (quadratic)	−0.0731
CoM	Phase × Load	0.0599	[0.0013, 0.1121]	0.982	Positive	log10(m^2^)	CoM	Phase × Load	0.0599
CoM	Between-subject SD	0.8281	[0.1671, 1.7208]	1.000	N/A (variance)	log10(m^2^)	CoM	Between-subject SD	0.8281

Note: HDI = Highest Density Interval (95% credibility, narrowest interval containing 95% of posterior mass). P = Posterior probability that the parameter exceeds zero (directional evidence). Evidence categories: Strong + (*p* > 0.99), Positive (*p* > 0.95), Uncertain (0.05 < *p* < 0.95), Negative (*p* < 0.05). All priors: Unified adaptive Empirical Bayes with shrinkage = 0.8 toward the literature anchors [2,6,7,14,15]. Intercept units: log10(m^2^); Phase/Load effects: log10(m^2^) per unit change; Load linear: per 30% 1 RM change; Load quadratic: per (30% 1 RM) 2. Between-subject SD: standard deviation of random subject intercepts. MCMC: NUTS, 4 chains × 2000 draws, 1000 tuning iterations, target_accept = 0.95. Convergence verified: R-hat < 1.01, ESS > 1000 for all reported parameters. Software: PyMC 5.16, ArviZ 0.19, Python 3.11.

**Table A4:** Data Validation Log for Ellipse Area Calculations.

Timestamp	Metric	Issue Type	Severity	Value	Details
2026-06-09T06:17:22.120195	Govea_CoPt_20_4	ABOVE_PHYSICAL_THRESHOLD	WARNING	0.0355	Area exceeds reasonable maximum
2026-06-09T06:17:22.122681	Govea_CoPpd_20_4	ABOVE_PHYSICAL_THRESHOLD	WARNING	0.0164	Area exceeds reasonable maximum
2026-06-09T06:17:22.123914	Govea_CoPt_35_1	ABOVE_PHYSICAL_THRESHOLD	WARNING	0.0106	Area exceeds reasonable maximum
2026-06-09T06:17:22.132816	Govea_CoPt_35_4	ABOVE_PHYSICAL_THRESHOLD	WARNING	0.0240	Area exceeds reasonable maximum
2026-06-09T06:17:22.159200	Govea_CoPt_80_1	ABOVE_PHYSICAL_THRESHOLD	WARNING	0.0116	Area exceeds reasonable maximum
2026-06-09T06:17:22.166773	Govea_CoPt_80_4	ABOVE_PHYSICAL_THRESHOLD	WARNING	0.0107	Area exceeds reasonable maximum
2026-06-09T06:17:28.614991	Leon_CoPt_20_4	ABOVE_PHYSICAL_THRESHOLD	WARNING	0.0104	Area exceeds reasonable maximum
2026-06-09T06:17:28.626748	Leon_CoPt_35_4	ABOVE_PHYSICAL_THRESHOLD	WARNING	0.0150	Area exceeds reasonable maximum
2026-06-09T06:17:28.644428	Leon_CoPt_65_1	ABOVE_PHYSICAL_THRESHOLD	WARNING	0.0104	Area exceeds reasonable maximum
2026-06-09T06:17:28.652661	Leon_CoPt_65_4	ABOVE_PHYSICAL_THRESHOLD	WARNING	0.0102	Area exceeds reasonable maximum
2026-06-09T06:17:28.663991	Leon_CoPt_80_4	ABOVE_PHYSICAL_THRESHOLD	WARNING	0.0164	Area exceeds reasonable maximum
2026-06-09T06:17:33.414695	Lugo_CoPt_65_4	ABOVE_PHYSICAL_THRESHOLD	WARNING	0.0125	Area exceeds reasonable maximum
2026-06-09T06:17:33.420890	Lugo_CoPt_80_1	ABOVE_PHYSICAL_THRESHOLD	WARNING	0.0172	Area exceeds reasonable maximum
2026-06-09T06:17:33.434458	Lugo_CoPt_80_4	ABOVE_PHYSICAL_THRESHOLD	WARNING	0.0143	Area exceeds reasonable maximum

Note: Severity levels: ERROR = data excluded from analysis; WARNING = data retained with caution; INFO = advisory. MIN_POINTS_ELLIPSE = 30 points minimum for ellipse calculation. MIN_STD_THRESHOLD = 1 × 10^−5^ m (zero variability safeguard). MAX_RANGE_THRESHOLD = 2.0 m (calibration error detection). MIN_COP_AREA_PHYSICAL = 1 × 10^−6^ m^2^ (lower physical bound). MAX_COP_AREA_PHYSICAL = 1 × 10^−2^ m^2^ (upper reasonable bound).

## References

[1] Winter D.A. (1995). Human Balance and Posture Control during Standing and Walking. Gait Posture.

[2] Rodal M., Arrayales-Millán E.M., Gonzalez-Macías M.E., Pérez-Gómez J., Gianikellis K. (2025). Half Squat Mechanical Analysis Based on PBT Framework. Bioengineering.

[3] Goodman W.W., Helms E., Graham D.F. (2023). Individual Muscle Contributions to the Acceleration of the Center of Mass During the Barbell Back Squat in Trained Female Subjects. J. Strength Cond. Res..

[4] Palmieri R.M., Ingersoll C.D., Stone M.B., Krause B.A. (2002). Center-of-Pressure Parameters Used in the Assessment of Postural Control. J. Sport Rehabil..

[5] Sohn J., Koo D. (2023). Effects of Load Increase on Lower Extremity Kinetic and Kinematic Variables in the Back Squat Exercise. Technol. Health Care.

[6] Giustino V., Vicari D.S.S., Patti A., Figlioli F., Thomas E., Schifaudo N., Tedesco M., Drid P., Paoli A., Palma A. (2024). Postural Control during the Back Squat at Different Load Intensities in Powerlifters and Weightlifters. Ann. Med..

[7] Rodal M., Arrayales-Millán E.M., González-Macías M.E., Espinosa-Mogollón L., Pérez-Gómez J., Gianikellis K. (2026). Phase-Specific Joint Mechanical Power Contribution to the Half Squat Exercise: A PBT Framework Analysis. Appl. Sci..

[8] Chen D., Sun D., Li F., Wang D., Zhou Z., Gao Z., Gu Y. (2025). Identifying the Primary Kinetic Factors Influencing the Anterior–Posterior Center of Mass Displacement in Barbell Squats: A Factor Regression Analysis. Sensors.

[9] Duarte M., Zatsiorsky V.M. (2002). Effects of Body Lean and Visual Information on the Equilibrium Maintenance during Stance. Exp. Brain Res..

[10] Choi A., Kang T.G., Mun J.H. (2016). Biomechanical Evaluation of Dynamic Balance Control Ability During Golf Swing. J. Med. Biol. Eng..

[11] Hannan K.B., King A.C. (2022). Lower Limb Ground Reaction Force and Center of Pressure Asymmetry During Bodyweight Squats. Int. J. Sports Phys. Ther..

[12] Genthon N., Rougier P. (2005). Influence of an Asymmetrical Body Weight Distribution on the Control of Undisturbed Upright Stance. J. Biomech..

[13] Isableu B., Hlavackova P., Diot B., Vuillerme N. (2017). Regularity of Center of Pressure Trajectories in Expert Gymnasts during Bipedal Closed-Eyes Quiet Standing. Front. Hum. Neurosci..

[14] Quijoux F., Nicolaï A., Chairi I., Bargiotas I., Ricard D., Yelnik A., Oudre L., Bertin-Hugault F., Vidal P.P., Vayatis N. (2021). A Review of Center of Pressure (COP) Variables to Quantify Standing Balance in Elderly People: Algorithms and Open-Access Code*. Physiol. Rep..

[15] Schubert P., Kirchner M. (2014). Ellipse Area Calculations and Their Applicability in Posturography. Gait Posture.

[16] Ishida T., Samukawa M., Endo D., Kasahara S., Tohyama H. (2022). Effects of Changing Center of Pressure Position on Knee and Ankle Extensor Moments During Double-Leg Squatting. J. Sports Sci. Med..

[17] Swinton P.A., Lloyd R., Keogh J.W.L., Agouris I., Stewart A.D. (2012). A Biomechanical Comparison of the Traditional Squat, Powerlifting Squat, and Box Squat. J. Strength Cond. Res..

[18] Bakhshinejad J.A., Ramer J.D., Dunsmore K.A., Pelton L.M., Berglund L. (2025). Effects of Intensity and Fatigue on the Kinetics and Kinematics of the Barbell Squat, Bench Press, and Deadlift in Experienced Lifters: A Systematic Review. Sports Med. Open.

[19] Kruschke J.K. (2013). Bayesian Estimation Supersedes the t Test. J. Exp. Psychol. Gen..

[20] Gelman A., Rohilla S.C. (2013). Philosophy and the Practice of Bayesian Statistics. Br. J. Math. Stat. Psychol..

[21] World Medical Association (2025). World Medical Association Declaration of Helsinki: Ethical Principles for Medical Research Involving Human Participants.

[22] Aleksic J., Mesaroš D., Kanevsky D., Knežević O.M., Cabarkapa D., Faj L., Mirkov D.M. (2024). Advancing Field-Based Vertical Jump Analysis: Markerless Pose Estimation vs. Force Plates. Life.

[23] Federolf P., Kühne M., Schiel K., Reimeir E., Debertin D., Calisti M., Mohr M. (2025). Validation of Markerless (Theia3D^TM^) against Marker-Based (Vicon^TM^) Motion Capture Data of Postural Control Movements Analyzed through Principal Component Analysis. J. Biomech..

[24] Pfister A., West A.M., Bronner S., Noah J.A. (2014). Comparative Abilities of Microsoft Kinect and Vicon 3D Motion Capture for Gait Analysis. J. Med. Eng. Technol..

[25] Carvalho C., Serrão F.V., Martinez A.F., Da Silva Serrão P.R.M. (2024). Three-Dimensional Kinematics of the Trunk, Pelvis, Hip, and Knee during the Single-Leg Squat and Hip Torque in Subjects with Isolated Patellofemoral Osteoarthritis Compared to Individually Matched Controls: Preliminary Results. Arch. Rheumatol..

[26] Dempster T.W. (1955). The Anthropometry of Body Action—Annals New York Academy of Sciences. Ann. N.Y. Acad. Sci..

[27] Ernesti J., Kaiser P. (2025). Python 3: The Comprehensive Guide.

[28] Terry K., Gade V.K., Allen J., Forrest G.F., Barrance P., Thomas Edwards W. (2011). Cross-Correlations of Center of Mass and Center of Pressure Displacements Reveal Multiple Balance Strategies in Response to Sinusoidal Platform Perturbations. J. Biomech..

[29] Steiger J.H. (1980). Tests for Comparing Elements of a Correlation Matrix. Psychol. Bull..

[30] Button K.S., Ioannidis J.P.A., Mokrysz C., Nosek B.A., Flint J., Robinson E.S.J., Munafò M.R. (2013). Power Failure: Why Small Sample Size Undermines the Reliability of Neuroscience. Nat. Rev. Neurosci..

[31] Salvatier J., Wiecki T.V., Fonnesbeck C. (2016). Probabilistic Programming in Python Using PyMC3. PeerJ Comput. Sci..

[32] Kumar R., Carroll C., Hartikainen A., Martin O. (2019). ArviZ a Unified Library for Exploratory Analysis of Bayesian Models in Python. J. Open Source Softw..

[33] Vallat R. (2018). Pingouin: Statistics in Python. J. Open Source Softw..

[34] Casella G. (1985). An Introduction to Empirical Bayes Data Analysis. Am. Stat..

[35] Morris C.N. (1983). Parametric Empirical Bayes Inference: Theory and Applications. J. Am. Stat. Assoc..

[36] Flanagan S.P., Salem G.J. (2007). Bilateral Differences in The Net Joint Torques During The Squat Exercise. J. Strength Cond. Res..

[37] Flanagan S.P., Salem G.J. (2008). Lower Extremity Joint Kinetic Responses to External Resistance Variations. J. Appl. Biomech..

[38] Dionisio V.C., Almeida G.L., Duarte M., Hirata R.P. (2008). Kinematic, Kinetic and EMG Patterns during Downward Squatting. J. Electromyogr. Kinesiol..

[39] Hof A.L., Gazendam M.G.J., Sinke W.E. (2005). The Condition for Dynamic Stability. J. Biomech..

[40] Alighanbari M., Alighanbari S., Griffin L. (2025). Enclosing 95% Confidence Area and Volume to Center of Pressure and Center of Mass in Posturography Using Optimization Algorithm and Coordinate Ascent Methods. Comput. Biol. Med..

[41] Sadeghi H., Allard P., Prince F., Labelle H. (2000). Symmetry and Limb Dominance in Able-Bodied Gait: A Review. Gait Posture.

[42] Krawczyk-Suszek M., Martowska B., Sapuła R. (2022). Analysis of the Stability of the Body in a Standing Position When Shooting at a Stationary Target―A Randomized Controlled Trial. Sensors.

[43] Choi A., Sim T., Mun J.H. (2016). Improved Determination of Dynamic Balance Using the Centre of Mass and Centre of Pressure Inclination Variables in a Complete Golf Swing Cycle. J. Sports Sci..

[44] Koo T.K., Li M.Y. (2016). A Guideline of Selecting and Reporting Intraclass Correlation Coefficients for Reliability Research. J. Chiropr. Med..

